# Tuning the Coherent Propagation of Organic Exciton‐Polaritons through the Cavity Q‐factor

**DOI:** 10.1002/advs.202302650

**Published:** 2023-10-11

**Authors:** Ruth H. Tichauer, Ilia Sokolovskii, Gerrit Groenhof

**Affiliations:** ^1^ Departamento de Física Teórica de la Materia Condensada and Condensed Matter Physics Center (IFIMAC) Universidad Autónoma de Madrid Madrid E‐28049 Spain; ^2^ Nanoscience Center and Department of Chemistry University of Jyväskylä P.O. Box 35, 40014 Jyväskylä Finland

**Keywords:** excitation energy transfer, Fabry–Pérot cavity, molecular dynamics, polariton, strong light–matter coupling

## Abstract

Transport of excitons in organic materials can be enhanced through polariton formation when the interaction strength between these excitons and the confined light modes of an optical resonator exceeds their decay rates. While the polariton lifetime is determined by the Q(uality)‐factor of the optical resonator, the polariton group velocity is not. Instead, the latter is solely determined by the polariton dispersion. Yet, experiments suggest that the Q‐factor also controls the polariton propagation velocity. To understand this observation, the authors perform molecular dynamics simulations of Rhodamine chromophores strongly coupled to Fabry–Pérot cavities with various Q‐factors. The results suggest that propagation in the aforementioned experiments is initially dominated by ballistic motion of upper polariton states at their group velocities, which leads to a rapid expansion of the wavepacket. Cavity decay in combination with non‐adiabatic population transfer into dark states, rapidly depletes these bright states, causing the wavepacket to contract. However, because population transfer is reversible, propagation continues, but as a diffusion process, at lower velocity. By controlling the lifetime of bright states, the Q‐factor determines the duration of the ballistic phase and the diffusion coefficient in the diffusive regime. Thus, polariton propagation in organic microcavities can be effectively tuned through the Q‐factor.

## Graphical abstract

Polariton mediated exciton transport is investigated through atomistic molecular dynamics simulations of organic chromophores strongly coupled to a Fabry–Pérot microcavity. The simulation results suggest that the unexpected quality factor dependence of polariton propagation velocities in organic media stems from propagation and decay of highly photonic states, the lifetime of which is finely tuned by the quality factor of the cavity.

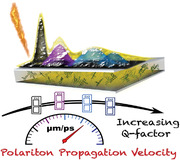

## 1 Introduction

Achieving long‐range energy transfer in organic media is a key requirement for enhancing the efficiency of opto‐electronic devices, such as organic diodes or solar cells, in which energy transport is limited by the incoherent diffusion mechanism that governs the motion of Frenkel excitons through materials. Recent experiments suggest that strongly coupling such excitons to the confined, but “delocalized”, modes of an optical resonator (called a cavity in what follows) can enhance transport through hybridization of the molecular excitons with the confined light modes into polaritons.^[^ ^1^, ^2^, ^3^, ^4^, ^5^, ^6^, ^7^, ^8^, ^9^, ^10^, ^11^, ^12^, ^13^, ^14^, ^15^ ^]^

**Figure 1:**
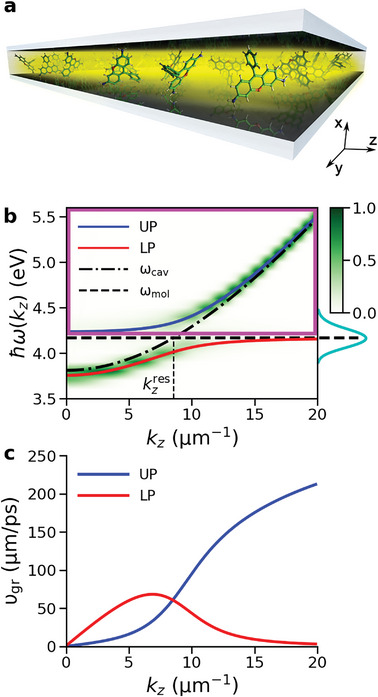
a) Schematic illustration of an optical Fabry‐Pérot micro‐cavity filled with Rhodamine chromophores (not to scale). b) Normalised angle‐resolved absorption spectrum of the cavity, showing Rabi splitting between the lower polariton (LP, red line) and the upper polariton (UP, blue line) branches. The cavity dispersion and absorption maximum of the molecules (4.18 eV at the CIS/3‐21G//Amber03 level of theory) are plotted by point‐dashed and dashed lines, respectively. The cyan line on the vertical axis depicts the absorption spectrum of Rhodamine. The purple frame encloses the range of polaritonic states excited instantaneously by the broad‐band pump pulse. c) Group velocity of the LP (red) and UP (blue), defined as ∂ω(*k*_*z*_)/∂*k*_*z*_.

Polaritons are coherent superpositions of molecular and cavity mode excitations that form when the interaction (*g*) between molecular excitons and cavity modes exceeds their decay rates (κ_mol_ and γ_cav_, respectively).^[^ ^16^, ^17^, ^18^ ^]^ The vast majority of these light–matter hybrid states have a negligible contribution from the cavity mode excitations and are therefore “dark”, forming a manifold of states, distributed around the molecular absorption maximum as illustrated in **Figure** **1**b. The fewer remaining states are bright and dispersive owing to their cavity mode contributions. They constitute the upper (UP) and lower polariton (LP) branches, also depicted in Figure 1b, that behave as quasi‐particles with low effective mass and large group velocity,^[^ ^19^ ^]^ defined as the derivative of the polariton energy with respect to *k*_*z*_‐vector (*v*_*g*_ = ∂ω/∂*k*_*z*_, Figure 1c). The low effective mass and large group velocity of polaritons can be exploited for controlled and long‐ranged *in‐plane* energy transport. Indeed, “in‐plane” polariton propagation has been observed in a variety of excitonic materials coupled to the confined light modes of Fabry‐Pérot cavities,^[^ ^7^, ^15^ ^]^ Bloch Surface Waves,^[^ ^5^, ^11^, ^20^ ^]^ Surface Lattice Resonances,^[^ ^14^ ^]^ and resonances arising from a dielectric constant mismatch between the excitonic medium and the surrounding environment.^[^ ^12^ ^]^

While these observations are in line with theoretical predictions,^[^ ^19^, ^21^, ^22^, ^23^, ^24^ ^]^ the propagation velocity observed in these experiments, is significantly lower than the group velocities inferred from the polariton dispersion (*v*_*g*_ = ∂ω/∂*k*_*z*_). In previous work,^[^ ^25^ ^]^ we used multi‐scale molecular dynamics (MD) simulations to resolve this discrepancy, and showed that irrespective of the initial excitation conditions, polariton propagation is a diffusion process on long timescales (> 100 fs). This diffusion is due to reversible population transfers between the stationary dark state manifold and the highly mobile bright polariton states, which render the propagation speed much slower than the polariton group velocities.^[^ ^14^, ^25^, ^26^ ^]^ Nevertheless, even if on longer timescales, propagation is not ballistic, polariton diffusion can significantly outperform exciton diffusion, which is typically limited to a few nanometers in organic materials.^[^ ^7^ ^]^

While we observed that cavity loss, caused by photon leakage through imperfect mirrors, reduces the distance over which polaritons propagate, we had not systematically investigated the effect of the cavity mode lifetime, $\tau_{\text{cav}}=\gamma_{\text{cav}}^{-1}$, which is related to the quality factor (Q‐factor) via Q = ω_cav_τ_cav_. The cavity mode lifetime, in combination with the molecular dephasing rate (κ_mol_), determines how strong the light‐matter interaction (*g*) needs to be for the molecule‐cavity system to enter the strong coupling regime (for which various criteria are commonly employed:^[^ ^18^ ^]^ i) *g* ⩾ γ_cav_, κ_mol_; ii) *g* ^2^ ⩾ (γ_cav_ − κ_mol_)^2^/4; iii) $g^{2}\geq \left(\gamma_{\text{cav}}^{2}+\kappa_{\text{mol}}^{2}\right)/2$; or iv) *g* ⩾ (γ_cav_ + κ_mol_)/2). Indeed, the Rabi splitting between the LP and UP branches ($\Omega^{\text{Rabi}}=2\sqrt{g^{2}N-\left(\gamma_{\text{cav}}-\kappa_{\text{mol}}\right){}^{2}/4}$, with *N* the number of molecules collectively coupled to the confined light modes, Figure 1) depends on both γ_cav_ and κ_mol_. Therefore, as shown in Figure S6 (Supporting Information, SI), the Q‐factor influences the Rabi splitting, but only marginally for systems that are well within the strong coupling regime. For such systems, the Q‐factor only influences the lifetime of organic polaritons, but not the light‐matter coupling strength.^[^ ^27^ ^]^ Yet, in recent femtosecond transient absorption microscopy (fs‐TAM) experiments on BODIPY‐R dyes in Fabry‐Pérot cavities with varying Q‐factors, Pandya et al. observed that the polariton propagation velocity can be enhanced by increasing the cavity Q‐factor.^[^ ^15^ ^]^ As emphasized by the authors, such “unexpected link between the Q‐factor and polariton velocity, is not captured by current models of exciton‐polaritons”.

To address this controversy and determine how the cavity Q‐factor influences the propagation of organic polaritons, we performed atomistic MD simulations of Rhodamine chromophores strongly coupled to the confined light modes of one‐dimensional (1D) uni‐directional Fabry‐Pérot cavities^[^ ^28^, ^29^ ^]^ with three different cavity mode lifetimes: τ_cav_ = 15, 30, and 60 fs. As before, the hydrated Rhodamines were modeled at the hybrid Quantum Mechanics / Molecular Mechanics (QM/MM) level.^[^ ^30^, ^31^ ^]^ We calculated mean‐field semi‐classical MD trajectories of 512 molecules, including their solvent environment, strongly coupled to the 160 confined light modes of a red‐detuned cavity (370 meV below the excitation energy of Rhodamine, which is 4.18 eV at the CIS/3‐21G//Amber03 level of theory employed here, see Computational Details and Supporting Information for details). Because in the fs‐TAM measurements of Pandya et al.^[^ ^15^ ^]^ the 10 fs broad‐band pump pulses populate mostly UP states, we modeled the initial excitation by preparing a Gaussian wavepacket of UP states centered at ℏω= 4.41 eV with a bandwidth of σ = 7.07 µm^−1^.^[^ ^19^ ^]^ The energy range of the states excited initially in this superposition is indicated by the magenta box in Figure 1b.

## 2 Results and Discussion

**Figure 2:**
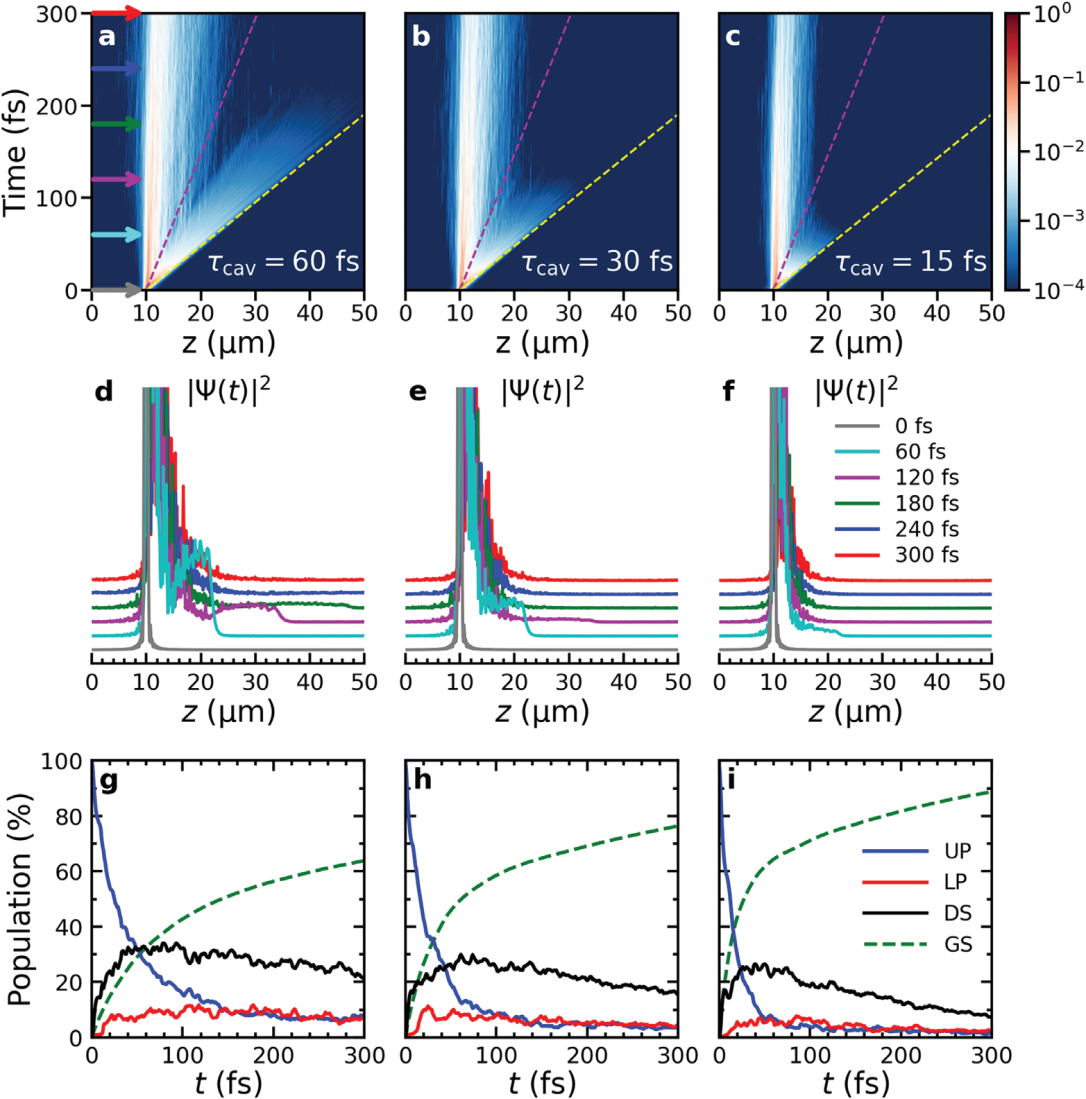
Polariton propagation after resonantly exciting a wavepacket of states in the UP branch centered at z=10 µm. a–c) Probability density of the total wave function, |Ψ(*t*)|^2^, as a function of distance (horizontal axis) and time (vertical axis) in cavities with different Q‐factors (i.e., τ_cav_ = 60, 30 and 15 fs, respectively). Colored arrows in panel a correspond to the time points of the 1D projection in panels (d–f). The dashed purple and yellow lines indicate propagation at the maximum group velocity of the LP (68 µm ps^−1^) and UP (212 µmps^−1^) branches, respectively. d–f) Probability density of the total polariton wave function, |Ψ(*t*)|^2^, at different time points as a function of distance. g–i) Populations of the UP (blue), LP (red), and dark (DS, black) states, as well as of the ground state (GS, green dashed line) as functions of time.

In **Figure** **2**, we show the time evolution of the probability density of the polaritonic wave function (|Ψ(*t*)|^2^, Equation 3), after instantaneous excitation of a Gaussian wavepacket of UP states in three Fabry‐Pérot microcavities supporting cavity modes with 15, 30, and 60 fs lifetimes, and containing 512 Rhodamine molecules. Animations of the propagation of the total, molecular and photonic wavepackets are provided as Supporting Information.

In all cavities the wavepacket initially broadens due to the wide range of UP group velocities. Around 30 fs, however, the wavepacket splits into (i) a faster component with a short lifetime that depends on the Q‐factor, and (ii) a slower component that is long‐lived, but almost stationary. While the lifetime of the slower component is hardly affected by the cavity lifetime, its broadening is Q‐factor dependent (Figure 2a–f). The long lifetime of the slower part suggests that it is composed mostly of dark states that lack group velocity, and into which some population of the initially excited UP states has relaxed. Nevertheless, due to thermally driven population transfer from these dark states back into propagating polaritons,^[^ ^32^ ^]^ the slower part still propagates. Because this transfer process is reversible and leads to transient occupation of polaritonic states over a wide range of *k*_*z*_‐vectors in both LP and UP branches, propagation occurs in a diffusive manner.^[^ ^14^, ^25^, ^26^ ^]^

In contrast, the faster component of the wavepacket is mainly composed of the higher‐energy UP states, which have high group velocity. Because the rate at which population transfers from these UP states into the dark state manifold is inversely proportional to the energy gap,^[^ ^33^ ^]^ the main decay channel for these states is radiative emission through the imperfect cavity mirrors. Thus, the lifetime and hence propagation distance of the faster wavepacket component is Q‐factor dependent, which is reflected by a faster rise of ground‐state population when the cavity mode lifetime decreases (green dashed lines in Figure 2g–i).

**Figure 3:**
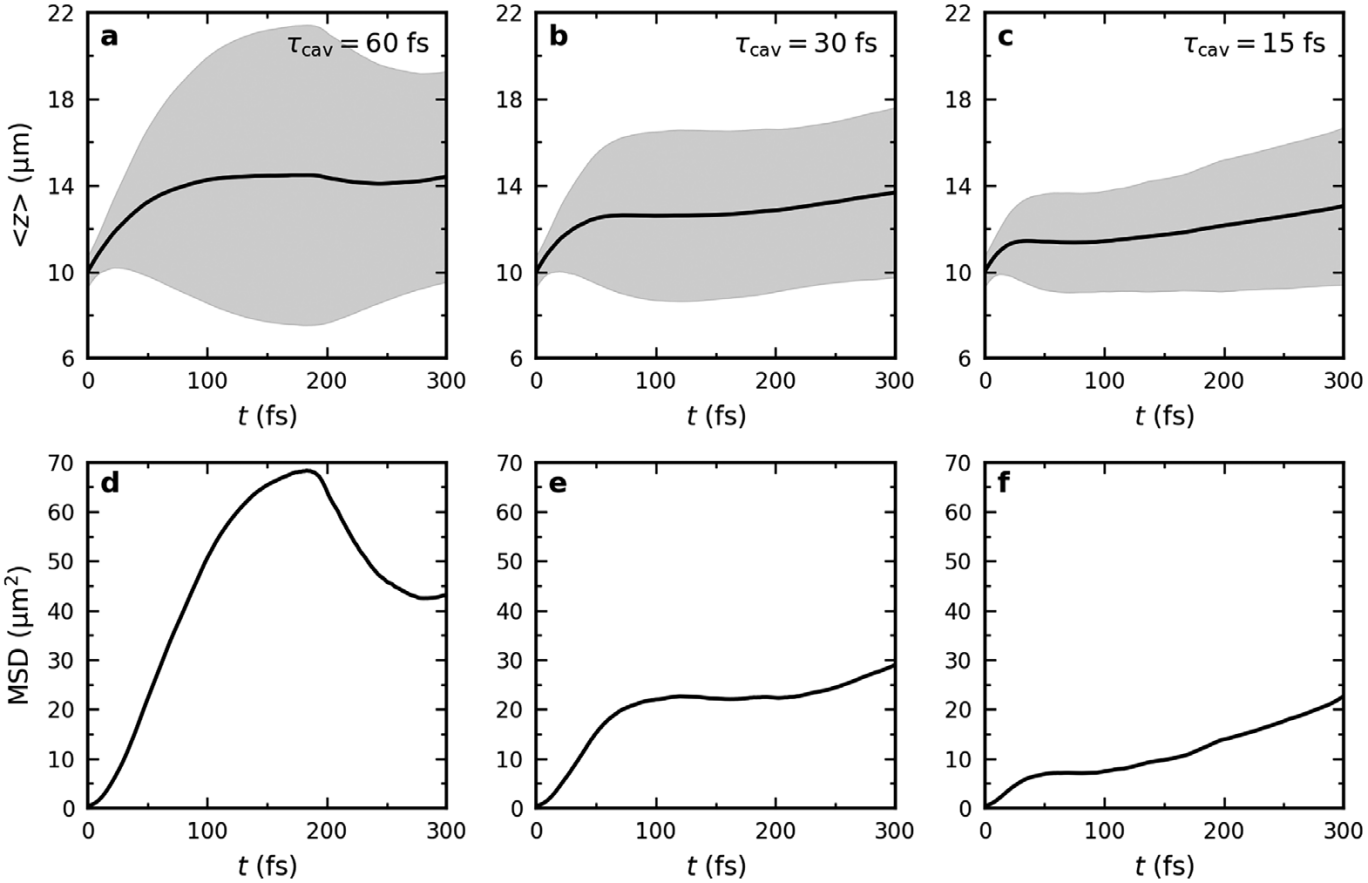
Top panels: Expectation value of the position of the total time‐dependent wavefunction $\left\langle z\right\rangle =\langle \Psi \left(t\right)|\hat{z}\left(t\right)|\Psi \left(t\right)\rangle /\left\langle \Psi \left(t\right)|\Psi \left(t\right)\right\rangle$ after on‐resonant excitation of UP states in cavities with different Q‐factors (i.e., τ_cav_ = 60 (left), 30 (middle) and 15 fs (right). The black lines represent 〈*z*〉 while the shaded areas indicate the root mean squared deviation (RMSD, i.e., $\sqrt{\left\langle \left(z\left(t\right)-\left\langle z\left(t\right)\right\rangle \right){}^{2}\right\rangle }$). Bottom panels: Mean squared displacement (MSD, i.e., $\left\langle \Psi \left(t\right)\right|\left(\hat{z}\left(t\right)-\hat{z}\left(0\right)\right){}^{2}\left|\Psi \left(t\right)\right\rangle /\left\langle \Psi \left(t\right)|\Psi \left(t\right)\right\rangle$) in the same cavities.

After the rapid initial expansion of the total wavepacket due to the population in the UP states (blue lines in Figure 2g‐i), transfer into the dark states (black lines), in combination with irreversible radiative decay from states with the highest group velocity, causes the wavepacket to contract. The extent of this contraction as well as the moment at which it takes place, depends on the cavity mode lifetime, as indicated by both the position, <*z*>, and Mean Squared Displacement (MSD) of the wavepackets in **Figure** **3**.

Whereas during the expansion phase propagation is dominated by ballistic motion of fast UP states that reach longer distances for higher Q‐factors (or equivalently, higher cavity mode lifetimes τ_cav_), as indicated by the maximum of the MSD (∽68, 23, and 7 µm^2^), after contraction, propagation continues as diffusion which is indicated by the linearity of the MSD at the end of the simulations (Figure 3). Diffusion emerges as a consequence of reversible population transfers between stationary dark states and mobile bright states at all *k*_*z*_‐vectors in both the UP and LP branches.^[^ ^25^ ^]^ The turnover from ballistic propagation into diffusion is Q‐factor dependent and occurs later when the cavity mode lifetime is higher (Figure 3).

While simulations provide detailed mechanistic insights into polariton propagation, direct observation of such details is challenging experimentally, in particular because the multiple contributions to a single transient spectral signal of a molecule‐cavity system cannot always be unambiguously disentangled.^[^ ^34^ ^]^ In their fs‐TAM experiments, Pandya et al.^[^ ^15^ ^]^ monitored the propagation of the wavepacket, Ψ(*z*, *t*), by probing transient changes in cavity transmission at a wavelength that is sensitive to LP absorption. As explained in the SI, to mimic such pump‐probe conditions in our simulations, we extracted position‐dependent transient changes in the transmission from our trajectories as follows:

(1) $$\frac{\Delta T(z,t)}{T_{0}}=\exp\left(\varepsilon_{a}d{\left|\Psi \left(z,t\right)\right|}^{2}\right)-1$$

with Δ*T*(*z*, *t*) = *T*(*z*, *t*) − *T*_0_ the difference between *T*(*z*, *t*), the transmission at position *z* and time *t* after excitation, and *T*_0_ = *T*(*z*, 0), the transmission before excitation. The variable ɛ_a_ is the absorption coefficient and *d* the path length. Because the value of ε_a_ cannot be derived directly from MD simulations, we treated it together with *d* as a single parameter. Here, we used ε_a_ *d*= 0.5, but, as we show in SI, varying this parameter does not change the results qualitatively. As was done in experiments,^[^ ^7^, ^15^, ^20^ ^]^ we characterize the propagation of the total wavepacket by the MSD of the transient signal, in our case of the transient transmission (Δ*T*/*T*_0_, Equation 1):

(2) $$\begin{array}{ccc} {\text{MSD}}_{T} & = & \sum_{i}^{N}{\left(z_{i}-z_{0}\right)}^{2}\frac{\Delta T(z,t)}{T_{0}}\\ & = & \sum_{i}^{N}{\left(z_{i}-z_{0}\right)}^{2}\left[\exp\left(\varepsilon_{a}d{\left|\Psi \left(z_{i},t\right)\right|}^{2}\right)-1\right] \end{array}$$

with *z*_0_ the expectation value of the position of the wavepacket at the start of the simulation (*t* = 0) and the sum is over the positions *z*_*i*_ of the *N* = 512 molecules. Full details of this analysis are provided in SI.

**Figure 4:**
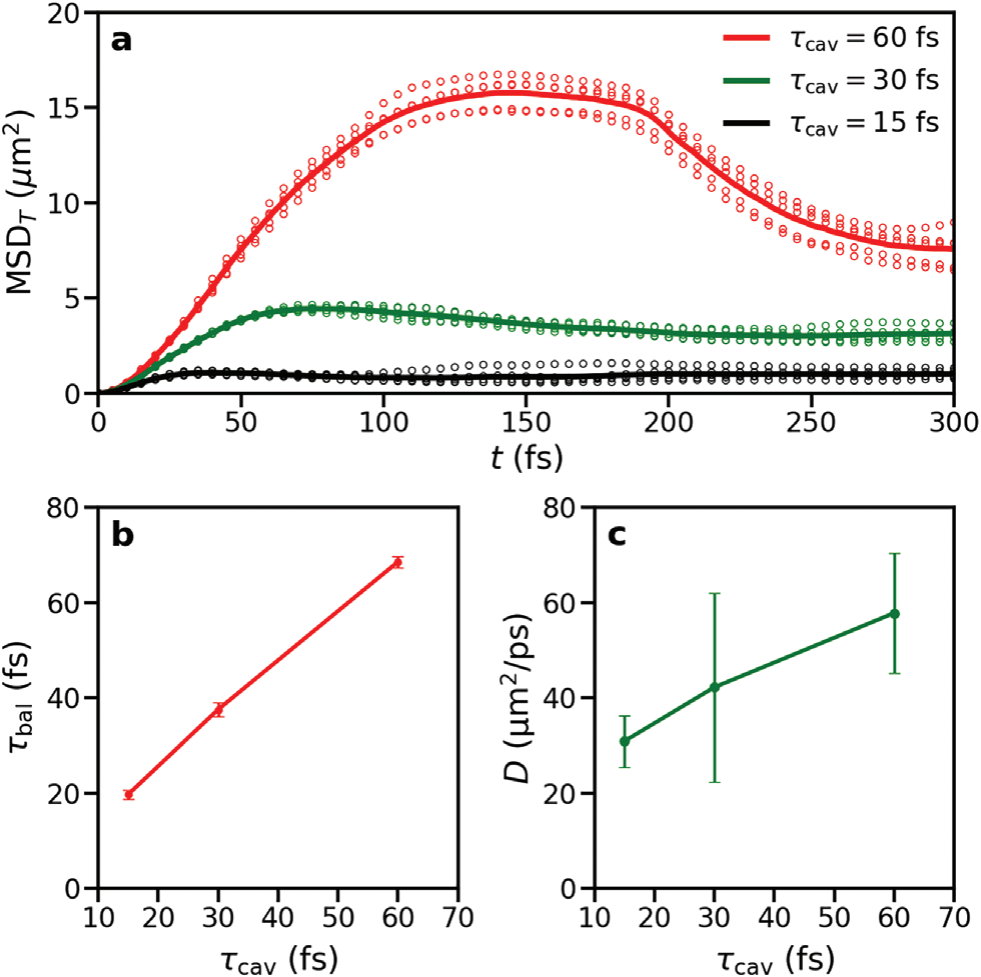
a) Mean squared displacement of the transmission signal (MSD_*T*_) for different cavity mode lifetimes: τ_cav_ = 60 (red), 30 (green) and 15~fs (black). Circles represent data points for individual runs, while the curves show the averages over all trajectories (five for each τ_cav_). b) The duration of the ballistic phase as a function of cavity mode lifetime. c) The diffusion coefficient in the diffusion regime as a function of cavity mode lifetime.

In **Figure** **4**a, we plot the MSD_*T*_ of the transient differential transmission for our cavity systems. As in the experiments (Figure 2c in Pandya et al.^[^ ^15^ ^]^), we observe that after a rapid initial increase, the MSD_*T*_ of the signal decreases. Based on our simulations we attribute this observation to the fast expansion of the wavepacket followed by the contraction. Because two propagation regimes were observed in our simulations, we analyzed these regimes separately. In contrast, Pandya et al. assumed a single ballistic phase, and extracted the velocity and duration of that phase from a global fit to the full MSD_T_ of the measured Δ*T*/*T*_0_ signal.

Because in the initial stages of the ballistic regime (*t* < τ_cav_) propagation is dominated by the population in UP states with well‐defined dispersion, the propagation speed is independent of the Q‐factor and determined solely by the UP group velocity (Figure 1c) in all cavities (Figure S2b in SI). However, the duration of this ballistic regime, τ_bal_, extracted from the Δ*T*/*T*_0_ signal by fitting the same function as Pandya et al. to the initial rise of the MSD_*T*_ (SI), depends on the cavity lifetime, and lasts longer if the cavity Q‐factor is higher, as shown in Figure 4b. Therefore, as in the MSD plots of the total wavepacket in Figure 3, the MSD_*T*_ of the Δ*T*/*T*_0_ signal also reaches the highest value in the cavity with highest Q‐factor (or equivalently, the longest cavity mode lifetime τ_cav_), in line with the fs‐TAM measurements. Because the initial ballistic propagation is dominated by population in the higher energy UP states that, isolated from the dark state manifold, decay through the imperfect cavity mirrors rather than transferring into the dark state manifold due to the inverse scaling of the transfer rate with the energy gap,^[^ ^33^ ^]^ the start of the wavepacket contraction is related to the duration of the ballistic phase τ_bal_. Indeed, by varying the center of the wavepacket (SI), and hence the energy gap to the dark states, the time at which contraction occurs can be controlled. According to the function used for fitting the MSD_*T*_ data (Equation S28, SI), the maximum of MSD_*T*_ occurs at *t* = 2τ_bal_ ≈ 2τ_cav_. Therefore, the moment at which contraction of the total wavepacket begins is proportional to the cavity lifetime (Figure 4a).

Whereas in their model Pandya et al. consider only ballistic propagation on a sub‐ps timescale, our simulations suggest that also diffusion contributes to propagation on those timescales, when solely the slower part of the wavepacket remains. Therefore, to characterize also this regime, we calculated the diffusion coefficient by fitting the linear regime of the MSD (SI). However, because in the MSD_*T*_ of the transient transmission (Figure 4a), the linear regime is difficult to discern, we performed the linear fit to the MSD associated with the slower component of the wavepacket at the end of the trajectories (Figure S5, SI). In Figure 4c, we plot the diffusion coefficients as a function of cavity mode lifetime. Because the overall diffusion process is a sequence of ballistic propagation phases, interrupted by stationary phases, and the duration of the ballistic phases is determined by non‐adiabatic coupling, which sets the rate for reversible population transfer into the dark state manifold, in combination with cavity decay, which sets the rate for irreversible loss via the imperfect cavity mirrors, the diffusion coefficient depends on the Q‐factor and increases with cavity lifetime (Figure 4c).

Because in our simulations we cannot couple as many molecules to the cavity as in experiment (i.e., 10^5^–10^8^ molecules^[^ ^35^, ^36^, ^37^ ^]^), we overestimate the diffusion coefficient. As we could show previously,^[^ ^33^ ^]^ the rate of population transfer from dark to bright states is inversely proportional to *N*, whereas the rate in the opposite direction is independent of *N*. Therefore, the population in the bright propagating states is overestimated when only 512 molecules are coupled to the cavity, leading to a faster diffusion. The overestimation of the diffusion coefficient thus leads to a much more pronounced increase of the wavepacket MSD than in experiment, where the total population residing in the propagating states is significantly lower,^[^ ^20^ ^]^ and diffusion would be hardly observable on sub‐ps timescales. Nevertheless, despite these quantitative differences, our simulations provide a qualitative picture that is in line with experimental observations.^[^ ^15^ ^]^

The results of our simulations suggest that the cavity lifetime controls both the duration and length of the initial ballistic phase (Figure 4b) as well as the diffusion constant in the diffusive regime (Figure 4c). Thus, without affecting polariton group velocity, the cavity Q‐factor provides an effective means to tune energy transport in the strong coupling regime. Our results therefore provide a rationale for the link between the Q‐factor and the propagation velocity, reported by Pandya et al., that is based on a current model of exciton‐polaritons.^[^ ^29^, ^38^ ^]^

Because our model combines established approaches from Quantum Optics, Quantum Chemistry and Molecular Dynamics,^[^ ^29^, ^38^ ^]^ our explanation does not rely on additional assumptions beyond the approximations underlying those approaches. Our explanation for the observations of Pandya et al. is, however, quite different from theirs,^[^ ^15^ ^]^ who, by making the additional assumption that the overlap between the molecular absorption spectrum and the cavity line width, determines which molecules can couple to the cavity, proposed that increasing the cavity Q‐factor reduces the energetic disorder and thereby increases the delocalization of dark states.^[^ ^15^ ^]^ Because the rate of population transfer between the dark state manifold and the polaritonic states depends on wave function overlap,^[^ ^33^ ^]^ increasing the delocalization in the dark states is speculated to enhance the thermal population exchange with bright states,^[^ ^32^ ^]^ thereby regenerating the highly propagating polariton states. However, the assumption that the cavity line‐width determines which molecules couple seems to contrast previous findings that “Rabi splitting occurs from a collective contribution of the whole inhomogeneous band of electronic state and not from a sharp selection of the state exactly resonant with the photon mode”.^[^ ^35^ ^]^ Nevertheless, although our explanation requires fewer assumptions, additional fs‐TAM experiments, in which temperature and dye concentration are varied to control the heterogeneous absorption line width and non‐adiabatic coupling, are urgently needed to test the validity of both explanations. As the duration of the ballistic phase depend on cavity lifetime, increasing Q‐factor would reduce the time and spacial resolution required for observing the contraction and the transition into the diffusion regime upon resonantly pumping a wavepacket of well‐isolated UP states, and thus facilitate these new experiments. Moreover, as additional sets of simulations, in which we i) varied the exciton‐photon detuning of the fundamental cavity mode at *k*_*z*_ = 0 by 100 meV, and ii) used narrow‐band pulses to instantaneously excite wavepackets of UP states centered at different *k*_*z*_ vectors (SI), suggest, the duration of the ballistic phase and hence the moment at which the transition and contraction occur, are not very sensitive to such energy detuning of the cavity, but can be controlled by varying the energy and spectral range of the excitation pulse.

## 3 Conclusion

To summarize, we have investigated the effect of the cavity Q‐factor on polariton propagation by means of atomistic MD simulations. The results of our simulations suggest that after the initial ballistic expansion, the wavepacket contracts due to irreversible radiative decay of population from states with the highest group velocities. In line with experiments, we find that the Q‐factor determines the propagation velocity and distance of organic polaritons via their lifetimes without affecting group velocities. Our findings therefore resolve the unexpected correlation between Q‐factor and propagation velocity reported by Pandya et al.^[^ ^15^ ^]^ Our results furthermore underscore that to understand the mechanism of polariton propagation and interpret experiments, it is necessary to include: i) atomic details for the material; ii) multiple modes for cavity dispersion; iii) cavity decay; and iv) sufficiently many molecules to have dark states providing an exciton reservoir. In particular, treating the molecular degrees of freedom of many molecules is essential for observing wavepacket contraction that is caused by cavity loss in combination with reversible non‐adiabatic population transfer between propagating bright states and the stationary long‐lived dark state manifold. Our work suggests that an ab initio description of molecules in multi‐mode cavities could pave the way to systematically design or optimize polariton‐based devices for enhanced energy transport.

## 4 Computational Details

We performed mean‐field semi‐classical^[^ ^39^ ^]^ MD simulations of 512 Rhodamine chromophores with their solvent environment, strongly coupled to 1D Fabry‐Pérot cavities with different radiative lifetimes: τ_cav_ = 15 fs, 30 fs, and 60 fs. To model the interactions between the molecules and the confined light modes of the cavity, we used a Tavis‐Cummings Hamiltonian, in which the molecular degrees of freedom are included.^[^ ^29^, ^38^ ^]^ A brief description of our multi‐scale cavity MD approach is provided as Supporting Information.

In our simulations the Rhodamine molecules were modelled at the QM/MM level, with the QM region containing the fused ring system of the molecule (Figure S2, Supporting Information). The ground‐state electronic structure of the QM subsystem was described at the restricted Hartree‐Fock (HF) method in combination with the 3‐21G basis set,^[^ ^40^ ^]^ while the excited‐state electronic structure was modeled with Configuration Interaction, truncated at single electron excitations (CIS/3‐21G). The MM region, which contains the rest of the chromophore as well as the solvent (3684 water molecules), was modeled with the Amber03 force field^[^ ^41^ ^]^ in combination with the TIP3P water model.^[^ ^42^ ^]^ At this level of QM/MM theory, the excitation energy of the Rhodamine molecules is 4.18 eV.^[^ ^38^ ^]^ In previous work, we showed that despite the overestimation of the vertical excitation energy, the topology of the potential energy surfaces is not very sensitive to the level of theory for Rhodamine.^[^ ^32^ ^]^

The uni‐directional 1D cavity with a length of *L*_*z*_ = 50 µm, with *z* indicating the in‐plane direction (*L*_*x*_ = 163 nm is the distance between the mirrors and *x* thus indicates the out‐of‐plane direction, see Figure S1 in the SI), was red‐detuned by 370 meV with respect to the molecular excitation energy (4.18 eV at the CIS/3‐21G//Amber03 level of theory, dashed line in Figure 1b), such that at wave vector *k*_*z*_ = 0, the cavity resonance is ℏω_0_ = 3.81 eV. The cavity dispersion, $\omega_{\text{cav}}\left(k_{z}\right)=\sqrt{\omega_{0}^{2}+c^{2}k_{z}^{2}/n^{2}}$, was modelled with 160 modes (0 ⩽ *p* ⩽ 159 for *k*_*z*_ = 2π*p*/*L*_*z*_), with *c* the speed of light and *n* the refractive index. Here, we used *n* = 1. See Supporting Information for further details on the cavity model.

The Rhodamine molecules were placed with equal inter‐molecular distances on the *z*‐axis of the cavity. To maximize the collective light‐matter coupling strength, the transition dipole moments of the Rhodamine molecules were aligned to the vacuum field at the start of the simulation. The same starting coordinates were used for all Rhodamines, but different initial velocities were selected randomly from a Maxwell–Boltzmann distribution at 300 K.

With a cavity vacuum field strength of 0.36 MV cm^−1^ (0.0000707 au), the Rabi splitting, defined as the energy difference between the bright lower (LP) and upper polariton (UP) branches at the wave‐vector $k_{z}^{\text{res}}$ where the cavity dispersion matches the molecular excitation energy (Figure 1b), is ∽325 meV for all cavities (τ_cav_ = 15 fs, 30 fs, and 60 fs). While the choice for a 1D cavity model with only positive *k*_*z*_ vectors was motivated by the necessity to keep our simulations computationally tractable, it precludes the observation of elastic scattering events that would change the direction (i.e., in‐plane momentum, ℏ**k**) of propagation. Furthermore, with only positive *k*_*z*_ vectors, polariton motion is restricted to the +*z* direction, but we could show previously^[^ ^25^ ^]^ that this assumption does not affect the mechanism of the propagation process.

Ehrenfest MD trajectories were computed by numerically integrating Newton's equations of motion using a leap‐frog algorithm with a 0.1 fs timestep. The multi‐mode Tavis‐Cummings Hamiltonian (See Supporting Information) was diagonalized at each timestep to obtain the *N* + *n*_mode_ (adiabatic) polaritonic eigenstates |ψ^*m*^> and energies *E* ^*m*^. The total polaritonic wavefunction |Ψ(*t*)> was coherently propagated along with the classical degrees of freedom of the *N* molecules as a time‐dependent superposition of the *N* + *n*_mode_ time‐independent adiabatic polaritonic states:

(3) $$\left|\Psi \left(t\right)\right\rangle =\sum_{m}^{N+n_{\text{mode}}}c_{m}\left(t\right)\left|\psi^{m}\right\rangle$$

where *c*_*m*_(*t*) are the time‐dependent expansion coefficients of the time‐independent polaritonic eigenstates |ψ^*m*^> (SI). A unitary propagator in the *local* diabatic basis was used to integrate these coefficients,^[^ ^43^ ^]^ while the nuclear degrees of freedom of the *N* molecules evolve on the mean‐field potential energy surface. Results reported in this work were obtained as averages over five trajectories for each cavity lifetime. For all simulations we used Gromacs 4.5.3,^[^ ^44^ ^]^ in which the multi‐mode Tavis‐Cummings QM/MM model was implemented,^[^ ^29^ ^]^ in combination with Gaussian16.^[^ ^45^ ^]^ Further details of the simulations are provided in the Supporting Information.

## Conflict of Interest

The authors declare no conflict of interest.

## Supporting information

Supporting Information

Supporting Information

Supporting Information

Supporting Information

Supporting Information

Supporting Information

Supporting Information

Supporting Information

Supporting Information

Supporting Information

## Data Availability

The data that support the findings of this study are available from the corresponding author upon reasonable request.

## References

[1] T. Freixanet , B. Sermage , A. Tiberj , R. Planel , Phys. Rev. B 2000, 61, 7233.

[2] D. M. Coles , N. Somaschi , P. Michetti , C. Clark , P. G. Lagoudakis , P. G. Savvidis , D. G. Lidzey , Nat. Mater. 2014, 13, 712. 24793357 10.1038/nmat3950

[3] X. Zhong , T. Chervy , S. Wang , J. George , A. Thomas , J. A. Hutchison , E. Devaux , C. Genet , T. W. Ebbesen , Angew. Chem. Int. Ed. 2016, 55, 6202. 10.1002/anie.201600428 27072296

[4] X. Zhong , T. Chervy , L. Zhang , A. Thomas , J. George , C. Genet , J. A. Hutchison , T. W. Ebbesen , Angew. Chem. Int. Ed. 2017, 56, 9034. 10.1002/anie.201703539 PMC5575472 28598527

[5] G. Lerario , D. Ballarini , A. Fieramosca , A. Cannavale , A. Genco , F. Mangione , S. Gambino , L. Dominici , M. D. Giorgi , G. Gigli , D. Sanvitto , Light Sci. Appl. 2017, 6, e16212. 30167229 10.1038/lsa.2016.212 PMC6062184

[6] D. M. Myers , S. Mukherjee , J. Beaumariage , D. W. Snoke , Phys. Rev. B 2018, 98, 235302.

[7] G. G. Rozenman , K. Akulov , A. Golombek , T. Schwartz , ACS Photonics 2018, 5, 105.

[8] Y. Zakharko , M. Rother , A. Graf , B. Hähnlein , M. Brohmann , J. Pezoldt , J. Zaumseil , Nano Lett. 2018, 18, 4927. 29995428 10.1021/acs.nanolett.8b01733 PMC6089499

[9] K. Georgiou , P. Michetti , L. Gai , M. Cavazzini , Z. Shen , D. G. Lidzey , ACS Photonics 2018, 5, 258.

[10] B. Xiang , R. F. Ribeiro , M. Du , L. Chen , Z. Yang , J. Wang , J. Yuen‐Zhou , W. Xiong , Science 2020, 368, 665. 32381725 10.1126/science.aba3544

[11] S. Hou , M. Khatoniar , K. Ding , Y. Qu , A. Napolov , V. M. Menon , S. R. Forrest , Adv. Mater. 2020, 32, 2002127. 10.1002/adma.202002127 32484288

[12] R. Pandya , R. Y. S. Chen , Q. Gu , J. Sung , C. Schnedermann , O. S. Ojambati , R. Chikkaraddy , J. Gorman , G. Jacucci , O. D. Onelli , T. Willhammar , D. N. Johnstone , S. M. Collins , P. A. Midgley , F. Auras , T. Baikie , R. Jayaprakash , F. Mathevet , R. Soucek , M. Du , A. M. Alvertis , A. Ashoka , S. Vignolini , D. G. Lidzey , J. J. Baumberg , R. H. Friend , T. Barisien , L. Legrand , A. W. Chin , J. Yuen‐Zhou , et al., Nat. Commun. 2021, 12, 6519. 34764252 10.1038/s41467-021-26617-w PMC8585971

[13] K. Georgiou , R. Jayaprakash , A. Othonos , D. G. Lidzey , Angew. Chem. Int. Ed. 2021, 60, 16661. 10.1002/anie.202105442 PMC8361947 33908681

[14] M. A. Berghuis , R. H. Tichauer , L. de Jong , I. Sokolovskii , P. Bai , M. Ramezani , S. Murai , G. Groenhof , J. G. Rivas , ACS Photonics 2022, 9, 123. 10.1021/acsphotonics.2c00007 PMC9306002 35880071

[15] R. Pandya , A. Ashoka , K. Georgiou , J. Sung , R. Jayaprakash , S. Renken , L. Gai , Z. Shen , A. Rao , A. J. Musser , Adv. Sci. 2022, 2105569. 10.1002/advs.202105569 PMC9218652 35474309

[16] P. Törmä , W. L. Barnes , Rep. Prog. Phys. 2015, 78, 013901. 25536670 10.1088/0034-4885/78/1/013901

[17] P. Forn‐Díaz , L. Lamata , E. Rico , J. Kono , E. Solano , Rev. Mod. Phys. 2019, 91, 025005.

[18] M. S. Rider , W. L. Barnes , Contemp. Phys. 2022, 62, 217.

[19] V. Agranovich , Y. Gartstein , Phys. Rev. B 2007, 75, 075302.

[20] M. Balasubrahmaniyam , A. Simkovich , A. Golombek , G. Ankonina , T. Schwartz , Nat. Mater. 2023, 22, 338. 36646793 10.1038/s41563-022-01463-3

[21] P. Michetti , G. C. La Rocca , Phys. Rev. B 2008, 77, 195301.

[22] M. Litinskaya , Phys. Lett. A 2008, 372, 3898.

[23] T. F. Allard , G. Weick , Phys. Rev. B 2022, 106, 245424.

[24] G. Engelhardt , J. Cao , Phys. Rev. B 2022, 105, 064205.

[25] I. Sokolovskii , R. H. Tichauer , D. Morozov , J. Feist , G. Groenhof , arXiv 2022, 2209.07309.

[26] D. Xu , A. Mandal , J. M. Baxter , S.‐W. Cheng , I. Lee , H. Su , S. Liu , D. R. Reichman , M. Delor , Nat. Commun. 2023, 14, 3881. 37391396 10.1038/s41467-023-39550-x PMC10313693

[27] L. Tropf , C. P. Dietrich , S. Herbst , A. L. Kanibolotsky , P. J. Skabara , F. Würthner , I. D. W. Samuel , M. C. Gather , S. Hoefling , Appl. Phys. Lett. 2017, 110, 153302.

[28] P. Michetti , G. C. L. Rocca , Phys. Rev. B. 2005, 71, 115320.

[29] R. Tichauer , J. Feist , G. Groenhof , J. Chem. Phys 2021, 154, 104112. 33722041 10.1063/5.0037868

[30] A. Warshel , M. Levitt , J. Mol. Biol. 1976, 103, 227. 985660 10.1016/0022-2836(76)90311-9

[31] M. Boggio‐Pasqua , C. F. Burmeister , M. A. Robb , G. Groenhof , Phys. Chem. Chem. Phys. 2012, 14, 7912. 22534732 10.1039/c2cp23628a

[32] G. Groenhof , C. Climent , J. Feist , D. Morozov , J. J. Toppari , J. Chem. Phys. Lett. 2019, 10, 5476. 10.1021/acs.jpclett.9b02192 PMC6914212 31453696

[33] R. H. Tichauer , D. Morozov , I. Sokolovskii , J. J. Toppari , G. Groenhof , J. Phys. Chem. Lett. 2022, 13, 6259. 35771724 10.1021/acs.jpclett.2c00826 PMC9289944

[34] S. Renken , R. Pandya , K. Georgiou , R. Jayaprakash , L. Gai , Z. Shen , D. G. Lidzey , A. Rao , A. J. Musser , J. Chem. Phys. 2021, 155, 154701. 34686047 10.1063/5.0063173

[35] R. Houdré , R. P. Stanley , M. Ilegems , Phys. Rev. A 1996, 53, 2711. 9913184 10.1103/physreva.53.2711

[36] E. Eizner , L. A. Martínez‐Martínez , J. Yuen‐Shou , S. Kéna‐Cohen , Sci. Adv. 2019, 5, eaax4484. 10.1126/sciadv.aax4482 PMC6897552 31840063

[37] L. A. Martínez‐Martínez , E. Eizner , S. Kéna‐Cohen , J. Yuen‐Zhou , J. Chem. Phys. 2019, 151, 054106. 10.1126/sciadv.aax4482 PMC6897552 31840063

[38] H.‐L. Luk , J. Feist , J. J. Toppari , G. Groenhof , J. Chem. Theory Comput. 2017, 13, 4324. 28749690 10.1021/acs.jctc.7b00388

[39] P. Ehrenfest , Z. Phys. 1927, 45, 445.

[40] R. Ditchfield , W. J. Hehre , J. A. Pople , J. Chem. Phys. 1971, 54, 724.

[41] Y. Duan , C. Wu , S. Chowdhury , M. C. Lee , G. M. Xiong , W. Zhang , R. Yang , P. Cieplak , R. Luo , T. Lee , J. Caldwell , J. M. Wang , P. Kollman , J. Comput. Chem. 2003, 24, 1999. 14531054 10.1002/jcc.10349

[42] W. L. Jorgensen , J. Chandrasekhar , J. D. Madura , R. W. Impey , M. L. Klein , J. Chem. Phys. 1983, 79, 926.

[43] G. Granucci , M. Persico , A. Toniolo , J. Chem. Phys. 2001, 114, 10608.

[44] B. Hess , C. Kutzner , D. van der Spoel , E. Lindahl , J. Chem. Theory Comput. 2008, 4, 435. 26620784 10.1021/ct700301q

[45] M. J. Frisch , G. W. Trucks , H. B. Schlegel , G. E. Scuseria , M. A. Robb , J. R. Cheeseman , G. Scalmani , V. Barone , G. A. Petersson , H. Nakatsuji , X. Li , M. Caricato , A. V. Marenich , J. Bloino , B. G. Janesko , R. Gomperts , B. Mennucci , H. P. Hratchian , J. V. Ortiz , A. F. Izmaylov , J. L. Sonnenberg , D. Williams‐Young , F. Ding , F. Lipparini , F. Egidi , J. Goings , B. Peng , A. Petrone , T. Henderson , D. Ranasinghe , et al., Gaussian 16 Revision C.01, Gaussian Inc., Wallingford CT 2016.

