# Supplementary material for: Tuning the Coherent Propagation of Organic Exciton‐Polaritons through the Cavity Q‐factor
Source: Adv Sci (Weinh). 2023 Oct 11;10(33):2302650. doi: 10.1002/advs.202302650 (PMC10667804; doi:10.1002/advs.202302650)
Supplement: Supplementary file 1 — Supporting Information [file ADVS-10-2302650-s003.pdf]

ADVANCED SCIENCE
Open Access

Supporting Information

for *Adv. Sci.*, DOI 10.1002/advs.202302650

Tuning the Coherent Propagation of Organic Exciton-Polaritons through the Cavity Q-factor

Ruth H. Tichauer, Ilia Sokolovskii and Gerrit Groenhof**

Supporting Information

for

Tuning the Coherent Propagation of Organic Exciton-Polaritons through the Cavity Q-factor

Ruth H. Tichauer,[*,†] Ilia Sokolovskii,[‡] and Gerrit Groenhof[‡]

†*Departamento de Física Teórica de la Materia Condensada and Condensed Matter Physics Center (IFIMAC), Universidad Autónoma de Madrid, Madrid, Spain.*

‡ *Nanoscience Center and Department of Chemistry, University of Jyväskylä, P.O. Box 35, 40014 Jyväskylä, Finland.*

E-mail: ruth.tichauer@uam.es

Contents

1 Molecular dynamics in the collective strong coupling regime **3**

1.1 Multi-scale Tavis-Cummings model 3

1.2 Multi-mode cavity model 4

1.3 Ehrenfest molecular dynamics simulations 7

1.4 Cavity decay 8

2 Further simulation details **9**

2.1 Rhodamine model 9

2.2 Initial conditions 10

3 Wavepacket analysis **11**

3.1 Populations of the lower polariton, upper polariton, and dark states 11

3.2 Wavepacket propagation 11

3.3 Transient transmission 13

3.4 Estimation of the duration and propagation velocity of the ballistic phase 14

3.5 Estimation of the diffusion coefficient in the diffusive phase 16

3.6 Influence of cavity decay rate on the Rabi splitting 16

4 Additional simulations **18**

4.1 Simulations with different cavity detunings 18

4.2 Propagation of narrow wavepackets 19

References **21**

1 Molecular dynamics in the collective strong coupling regime

1.1 Multi-scale Tavis-Cummings model

To model the dynamics of N dye molecules strongly coupled to n_{mode} confined light modes of a one-dimensional (1D) Fabry-Pérot cavity, we extended the Tavis-Cummings model[1,2] to account for both the molecular degrees of freedom,[3] and the cavity mode structure:[4]

$$\begin{aligned}\hat{H}^{\text{TC}} \;=\; & \textstyle\sum_j^N h\nu_j(\mathbf{R}_j)\hat{\sigma}_j^+\hat{\sigma}_j^- + \sum_{k_z}^{n_{\text{mode}}} \hbar\omega_{\text{cav}}(k_z)\hat{a}_{k_z}^\dagger\hat{a}_{k_z} + \\ & \textstyle\sum_j^N \sum_{k_z}^{n_{\text{mode}}} \hbar g_j(k_z)\left(\hat{\sigma}_j^+\hat{a}_{k_z}e^{ik_z z_j} + \hat{\sigma}_j^-\hat{a}_{k_z}^\dagger e^{-ik_z z_j}\right) + \\ & \textstyle\sum_i^N V_{\text{S}_0}^{\text{mol}}(\mathbf{R}_i)\end{aligned} \tag{1}$$

Here, $\hat{\sigma}_j^+$ ($\hat{\sigma}_j^-$) is the operator that excites (de-excites) molecule j from the electronic ground (excited) state $|\text{S}_0^j(\mathbf{R}_j)\rangle$ ($|\text{S}_1^j(\mathbf{R}_j)\rangle$) into the electronic excited (ground) state $|\text{S}_1^j(\mathbf{R}_j)\rangle$ ($|\text{S}_0^j(\mathbf{R}_j)\rangle$); $\mathbf{R}_j$ is the vector of the Cartesian coordinates of all atoms in molecule j, centered at z_j; $\hat{a}_{k_z}$ ($\hat{a}_{k_z}^\dagger$) is the annihilation (creation) operator of an excitation of a cavity mode with wave-vector k_z; $h\nu_j(\mathbf{R}_j)$ is the excitation energy of molecule j, defined as:

$$h\nu_j(\mathbf{R}_j) = V_{\text{S}_1}^{\text{mol}}(\mathbf{R}_j) - V_{\text{S}_0}^{\text{mol}}(\mathbf{R}_j) \tag{2}$$

with $V_{\text{S}_0}^{\text{mol}}(\mathbf{R}_j)$ and $V_{\text{S}_1}^{\text{mol}}(\mathbf{R}_j)$ the adiabatic potential energy surfaces of molecule j in the electronic ground (S_0) and excited (S_1) state, respectively.

The last term in Equation 1 is the total potential energy of the system in the absolute ground state (*i.e.*, with no excitations in neither the molecules nor the cavity modes), defined as the sum of the ground-state potential energies of all molecules in the cavity. The $V_{\text{S}_0}^{\text{mol}}(\mathbf{R}_j)$ and $V_{\text{S}_1}^{\text{mol}}(\mathbf{R}_j)$ adiabatic potential energy surfaces are modelled at the QM/MM level of theory,[5,6] as described in the Computational Details section of the main text.

The third term in Equation 1 models the light-matter interaction within the dipolar approximation through $g_j(k_z)$:

$$g_j(k_z) = -\boldsymbol{\mu}_j^{\text{TDM}}(\mathbf{R}_j) \cdot \mathbf{u}_{\text{cav}} \sqrt{\frac{\hbar\omega_{\text{cav}}(k_z)}{2\epsilon_0 V_{\text{cav}}}} \tag{3}$$

where $\boldsymbol{\mu}_j^{\text{TDM}}(\mathbf{R}_j)$ is the transition dipole moment of molecule j that depends on the molecular geometry ($\mathbf{R}_j$); $\mathbf{u}_{\text{cav}}$ the unit vector in the direction of the electric component of the cavity vacuum field (*i.e.*, $|\mathbf{E}| = \sqrt{\hbar\omega_{\text{cav}}(k_z)/2\epsilon_0 V_{\text{cav}}}$), chosen along the y-direction (see Figure S1); ϵ_0 the vacuum permittivity; and V_{cav} the cavity mode volume.

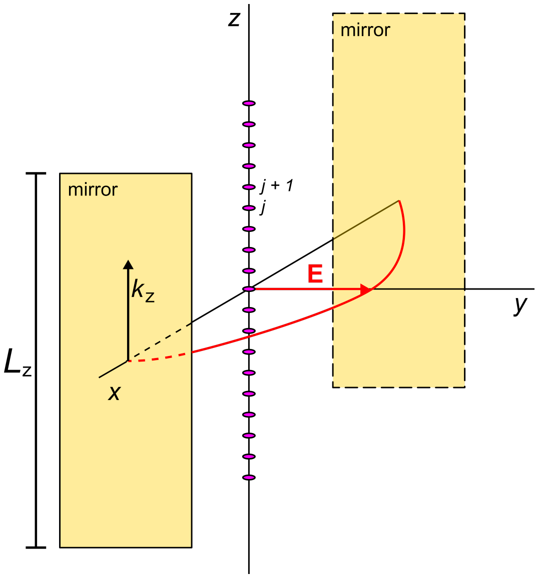

Figure S1: One-dimensional (1D) Fabry-Pérot micro-cavity model.[7] Two reflecting mirrors located at $-\frac{1}{2}x$ and $\frac{1}{2}x$, confine light modes along this direction, while free propagation along the z direction is possible for plane waves with in-plane momentum k_z and energy $\hbar\omega_{\text{cav}}(k_z)$. The vacuum field vector (red) points along the y-axis, reaching a maximum amplitude at $x = 0$ where the N molecules (magenta ellipses) are placed, distributed along the z-axis at positions z_j with $1 \leq j \leq N$.

1.2 Multi-mode cavity model

To discretize the cavity dispersion, we follow Michetti and La Rocca,[7] and impose periodic boundary conditions in the z-direction of the 1D cavity. Under these conditions, the wave vectors, k_z, adopt discrete values: $k_{z,p} = 2\pi p/L_z$ with $p \in \mathbb{Z}$ and L_z the length of the 1D cavity. After discretization the molecular Tavis-Cummings Hamiltonian in Equation 1 becomes a $(N + n_{\text{mode}})$ by $(N + n_{\text{mode}})$

matrix with four blocks:[4]

$$\mathbf{H}^{\mathrm{TC}} = \begin{pmatrix} \mathbf{H}^{\mathrm{mol}} & \mathbf{H}^{\mathrm{int}} \\ \mathbf{H}^{\mathrm{int}\dagger} & \mathbf{H}^{\mathrm{cav}} \end{pmatrix} \tag{4}$$

The elements of this matrix are evaluated in the product basis of adiabatic molecular states times cavity mode excitations:

$$\begin{aligned} |\phi_j\rangle &= \hat{\sigma}_j^{+}|\mathrm{S}_0^1\mathrm{S}_0^2..\mathrm{S}_0^{N-1}\mathrm{S}_0^N\rangle \otimes |00..0\rangle \\ &= \hat{\sigma}_j^{+}|\Pi_i^N \mathrm{S}_0^i\rangle \otimes |\Pi_k^{n_{\mathrm{mode}}} 0_k\rangle \\ &= \hat{\sigma}_j^{+}|\phi_0\rangle \end{aligned} \tag{5}$$

for $1 \leq j \leq N$, and

$$\begin{aligned} |\phi_{j>N}\rangle &= \hat{a}_{j-N}^{\dagger}|\mathrm{S}_0^1\mathrm{S}_0^2..\mathrm{S}_0^{N-1}\mathrm{S}_0^N\rangle \otimes |00..0\rangle \\ &= \hat{a}_{j-N}^{\dagger}|\Pi_i^N \mathrm{S}_0^i\rangle \otimes |\Pi_k^{n_{\mathrm{mode}}} 0_k\rangle \\ &= \hat{a}_{j-N}^{\dagger}|\phi_0\rangle \end{aligned} \tag{6}$$

for $N < j \leq N + n_{\mathrm{mode}}$. In these expressions $|00..0\rangle$ indicates that all Fock states associated with the n_{mode} cavity modes are empty. The basis state $|\phi_0\rangle$ is the ground state of the molecule-cavity system with no excitations in neither the molecules nor cavity modes:

$$|\phi_0\rangle = |\mathrm{S}_0^1\mathrm{S}_0^2..\mathrm{S}_0^{N-1}\mathrm{S}_0^N\rangle \otimes |00..0\rangle = |\Pi_i^N \mathrm{S}_0^i\rangle \otimes |\Pi_k^{n_{\mathrm{mode}}} 0_k\rangle \tag{7}$$

The upper left block, $\mathbf{H}^{\mathrm{mol}}$, is an $N \times N$ matrix that contains the single-photon excitations of the molecules. Because we neglect direct excitonic interactions between molecules, this block is

diagonal, with elements labeled by the molecule indices j:

$$H_{j,j}^{\text{mol}} = \langle \phi_0 | \hat{\sigma}_j \hat{H}^{\text{TC}} \hat{\sigma}_j^{+} | \phi_0 \rangle \tag{8}$$

for $1 \leq j \leq N$. Each matrix element of $\mathbf{H}^{\text{mol}}$ thus represents the potential energy of a molecule, j, in the electronic excited state $|\text{S}_1^j(\mathbf{R}_j)\rangle$ while all other molecules, $i \neq j$, are in the electronic ground state $|\text{S}_0^i(\mathbf{R}_i)\rangle$:

$$H_{j,j}^{\text{mol}} = V_{\text{S}_1}^{\text{mol}}(\mathbf{R}_j) + \sum_{i \neq j}^{N} V_{\text{S}_0}^{\text{mol}}(\mathbf{R}_i) \tag{9}$$

The lower right block in Equation 4, $\mathbf{H}^{\text{cav}}$, is an $n_{\text{mode}} \times n_{\text{mode}}$ matrix (with $n_{\text{mode}} = n_{\text{max}} - n_{\text{min}} + 1$) containing the single-photon excitations of the cavity modes, and is also diagonal:

$$H_{p,p}^{\text{cav}} = \langle \phi_0 | \hat{a}_p \hat{H}^{\text{TC}} \hat{a}_p^{\dagger} | \phi_0 \rangle \tag{10}$$

for $n_{\text{min}} \leq p \leq n_{\text{max}}$. Here, $\hat{a}_p^{\dagger}$ excites cavity mode p with wave-vector $k_{z,p} = 2\pi p / L_z$. In these matrix elements, all molecules are in the electronic ground state (S_0). The energy is therefore the sum of the cavity energy at $k_{z,p}$, and the molecular ground state energies:

$$H_{p,p}^{\text{cav}} = \hbar \omega_{\text{cav}}(2\pi p / L_z) + \sum_{j}^{N} V_{\text{S}_0}^{\text{mol}}(\mathbf{R}_j) \tag{11}$$

where, $\omega_{\text{cav}}(k_{z,p})$ is the cavity dispersion (dashed-dotted curve in Figure 1**b**, main text):

$$\omega_{\text{cav}}(k_{z,p}) = \sqrt{\omega_0^2 + c^2 k_{z,p}^2 / n^2} \tag{12}$$

with $\hbar\omega_0$ the energy at $k_{z,0} = 0$, n the refractive index of the medium and c the speed of light in vacuum.

The two $N \times n_{\text{mode}}$ off-diagonal blocks $\mathbf{H}^{\text{int}}$ and $\mathbf{H}^{\text{int}\dagger}$ in the multi-mode Tavis-Cummings Hamiltonian (Equation 4) model the light-matter interactions between the molecules and the cavity modes. These matrix elements are approximated as the inner product between the molecular

transition dipole moments on the one hand, and the transverse electric field of the cavity modes at the center of the molecules, on the other hand:

$$\begin{aligned} H_{j,p}^{\text{int}} &= -\boldsymbol{\mu}_j^{\text{TDM}}(\mathbf{R}_j)\cdot\mathbf{u}_{\text{cav}}\sqrt{\frac{\hbar\omega_{\text{cav}}(2\pi p/L_z)}{2\epsilon_0 V_{\text{cav}}}}\langle\phi_0|\hat{\sigma}_j(\hat{\sigma}_j^+\hat{a}_p e^{i2\pi p z_j/L_z})\hat{a}_p^\dagger|\phi_0\rangle \\ &= -\boldsymbol{\mu}_j^{\text{TDM}}(\mathbf{R}_j)\cdot\mathbf{u}_{\text{cav}}\sqrt{\frac{\hbar\omega_{\text{cav}}(2\pi p/L_z)}{2\epsilon_0 V_{\text{cav}}}}e^{i2\pi p z_j/L_z} \end{aligned} \tag{13}$$

for $1 \leq j \leq N$ and $n_{\text{min}} \leq p \leq n_{\text{max}}$.

Diagonalization of the multi-scale Tavis-Cummings Hamiltonian in Equation 4 yields the $N + n_{\text{mode}}$ hybrid light-matter states $|\psi^m\rangle$:[7,8]

$$|\psi^m\rangle = \left(\sum_j^N \beta_j^m \hat{\sigma}_j^+ + \sum_p^{n_{\text{mode}}} \alpha_p^m \hat{a}_p^\dagger\right)|\phi_0\rangle \tag{14}$$

with eigenenergies E_m. The expansion coefficients β_j^m and α_p^m reflect , respectively, the contribution of the molecular excitons ($|\text{S}_1^j(\mathbf{R}_j)\rangle$) and the cavity mode excitations ($|1_p\rangle$) to polariton $|\psi^m\rangle$.

1.3 Ehrenfest molecular dynamics simulations

MD trajectories of all molecules (including environment) were computed by numerically integrating Newton's equations of motion. The multi-mode Tavis-Cummings Hamiltonian (Equation 4) was diagonalized at each time-step of the simulation to obtain the $N + n_{\text{mode}}$ (adiabatic) polaritonic eigenstates $|\psi^m\rangle$ and energies E^m. The *total* polaritonic wavefunction $|\Psi(t)\rangle$ was coherently propagated along with the classical degrees of freedom of the N molecules as a time-dependent superposition of the $N + n_{\text{mode}}$ time-independent adiabatic polaritonic states:

$$|\Psi(t)\rangle = \sum_m^{N+n_{\text{mode}}} c_m(t)|\psi^m\rangle \tag{15}$$

where $c_m(t)$ are the time-dependent expansion coefficients of the time-independent eigenstates, $|\psi^m\rangle$, defined in Equation 14. A unitary propagator in the *local* diabatic basis was used to integrate

these coefficients,[9] while the nuclear degrees of freedom of the N molecules evolved on the mean-field potential energy surface:

$$V(\mathbf{R}) = \langle \Psi(t)|\hat{H}^{\text{TC}}|\Psi(t)\rangle \tag{16}$$

1.4 Cavity decay

Radiative loss through the imperfect cavity mirrors was modeled as a first-order decay process into the overall ground state of the system (*i.e.*, no excitation in neither the molecules nor the cavity modes).[3] Assuming that the intrinsic decay rates, γ_{cav}, are the same for all modes, the total loss rate was calculated as the product of γ_{cav} and the total photonic weight, $\sum_p^{n_{\text{mode}}} |\alpha_p^m|^2$, of state $|\psi^m\rangle$. Thus, after an MD step Δt, the population in state $|\psi_m\rangle$, $\rho_m(t) = |c_m(t)|^2$, becomes:

$$\rho_m(t+\Delta t) = \rho_m(t) \exp\left[-\gamma_{\text{cav}} \sum_p^{n_{\text{mode}}} |\alpha_p^m(t)|^2 \Delta t\right] \tag{17}$$

Since $\rho_m = (\Re[c_m])^2 + (\Im[c_m])^2$, changes in the real and imaginary parts of the (complex) expansion coefficients $c_m(t)$ due to spontaneous photonic loss through the mirrors of a low-Q cavity, are:

$$\Re[c_m(t+\Delta t)] = \Re[c_m(t)] \exp\left[-\frac{1}{2}\gamma_{\text{cav}} \sum_p^{n_{\text{mode}}} |\alpha_p^m(t)|^2 \Delta t\right]$$

$$\Im[c_m(t+\Delta t)] = \Im[c_m(t)] \exp\left[-\frac{1}{2}\gamma_{\text{cav}} \sum_p^{n_{\text{mode}}} |\alpha_p^m(t)|^2 \Delta t\right]$$

Simultaneously, the population of the zero-excitation subspace, or ground state, $\rho_0(t+\Delta t)$, increases as:

$$\rho_0(t+\Delta t) = \rho_0(t) + \sum_m \rho_m(t) \left(1 - \exp\left[-\gamma_{\text{cav}} \sum_p^{n_{\text{mode}}} |\alpha_p^m(t)|^2 \Delta t\right]\right) \tag{18}$$

2 Further simulation details

2.1 Rhodamine model

The Rhodamine molecules, one of which is shown schematically in Figure S2, were modelled with the Amber03 force field,[10] using the parameters provided by Luk *et al.*[3] After a geometry optimization at the force field level, the molecule was placed at the center of a rectangular box and 3,684 TIP3P water molecules,[11] were added. The simulation box, which contained 11,089 atoms, was equilibrated for 2 ns with harmonic restraints on the heavy atoms of Rhodamine (force constant 1000 $kJmol^{-1}nm^{-1}$). Subsequently, a 200 ns classical MD trajectory was computed at constant temperature (300 K) using a stochastic dynamics integrator with a friction coefficient of 0.1 ps^{-1}. The pressure was kept constant at 1 bar using the Berendsen isotropic pressure coupling algorithm[12] with a time constant of 1 ps. The LINCS algorithm was used to constrain bond lengths,[13] while SETTLE was applied to constrain the internal degrees of freedom of water molecules,[14] enabling a time step of 2 fs in the classical MD simulations. A 1.0 nm cut-off was used for Van der Waals' interactions, which were modeled with Lennard-Jones potentials. Coulomb interactions were computed with the smooth particle mesh Ewald method,[15] using a 1.0 nm real space cut-off and a grid spacing of 0.12 nm. The relative tolerance at the real space cut-off was set to 10^{-5}.

From the second half of the 200 ns MD trajectory, snapshots were extracted and subjected to further equilibration for 10 ps at the QM/MM level. The time step was reduced to 1 fs. As in previous work,[3,4,16] the fused ring system was included in the QM region and described at the RHF/3-21G level, while the rest of the molecule as well as the water solvent, were modelled with the Amber03 force field[10] and TIP3P water model,[11] respectively (Figure S2). The bond connecting the QM and MM subsystems was replaced by a constraint and the QM part was capped with a hydrogen atom. The force on the cap atom was distributed over the two atoms of the bond.[17] The QM system experienced the Coulomb field of all MM atoms within a 1.6 nm cut-off sphere and Lennard-Jones interactions between MM and QM atoms were added. The singlet electronic excited state (S_1) was modeled with the Configuration Interaction method, truncated at single electron

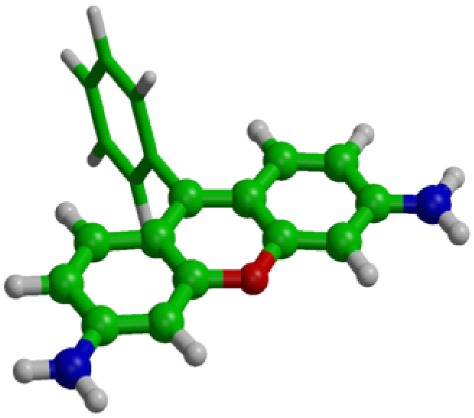

Figure S2: Rhodamine model used in our simulations. The QM subsystem, shown in ball-and-stick representation, is described at the HF/3-21G level of theory in the electronic ground state (S_0), and at the CIS/3-21G level of theory in the first singlet excited state (S_1). The MM subsystem, consisting of the atoms shown in stick representation, and the water molecules (not shown), are modelled with the Amber03 force field.

excitations, for the QM region (*i.e.,* CIS/3-21G//Amber03). At this level of QM/MM theory, the excitation energy is 4.18 eV[3]. The QM/MM simulations were performed with GROMACS version 4.5.3,[18] interfaced to TeraChem version 1.93.[19,20]

2.2 Initial conditions

The initial excitation of a polariton wavepacket was created by assigning to the expansion coefficients $c_m(t = 0)$ values of a Gaussian distribution centered at k-vector $k_c = 80\frac{2\pi}{L_z} = 10.05\ \mu\text{m}^{-1}$ and covering the whole UP branch (*i.e.*, excluding LP and dark states in the initial wavepacket):[8]

$$c_m(0) = \left(\frac{\zeta}{2\pi^3}\right)^{\frac{1}{4}} \exp[-\zeta(k_z^m - k_c)^2] \tag{19}$$

where $\zeta = 10^{-14}$ m^2 is a coefficient characterising the shape of the wavepacket and k_z^m the expectation value of the in-plane momentum of polariton $|\psi_m\rangle$, evaluated as:

$$\langle k_z^m \rangle = \frac{\sum_p^{n_{\text{mode}}} |\alpha_p^m|^2 k_{z,p}}{\sum_p^{n_{\text{mode}}} |\alpha_p^m|^2} \tag{20}$$

with $k_{z,p} = 2\pi p/L_z$ the discrete wave vector in a periodic 1D cavity of length L_z (see subsection 1.2).

3 Wavepacket analysis

3.1 Populations of the lower polariton, upper polariton, and dark states

The time evolution of the populations in the LP, UP, and dark states (plotted in panels **g**, **h**, **i** of Figure 2 in the main text) were obtained by summing over expansion coefficients that belong to LP, UP or dark states: *i.e.*, $\sum_m |c_m(t)|^2$, with $m \in$ LP (160 low-energy states), UP (160 higher-energy states), or dark states (the remaining states), respectively. With a Rabi splitting of $\sim$ 325 meV, the LP and UP branches are sufficiently separated from the dark state manifold for a correct assignment of all states in our simulations.

3.2 Wavepacket propagation

To monitor the propagation of the wavepackets, we plotted the probability density of the total time-dependent wave function $|\Psi(t)|^2$ at the positions of the molecules, z_j, as a function of time (panels **a–f** in figure 2 in the main text). We thus represent the density as a *discrete* distribution at grid points that correspond to the molecular positions, rather than as a continuous distribution.

The probability density of the total time-dependent wave function $|\Psi(t)|^2$ was calculated as the sum of the probability densities of the molecular $|\Psi_{\text{mol}}(t)|^2$ and photonic $|\Psi_{\text{pho}}(t)|^2$ contributions. The amplitude of $|\Psi_{\text{mol}}(t)\rangle$ at position z_j in the 1D cavity (with $z_j = (j-1)L_z/N$ for $1 \leq j \leq N$) was obtained by projecting the excitonic basis state in which molecule j at position z_j is excited, onto the total wave function (Equation 15):

$$\begin{aligned} |\Psi^{\text{mol}}(z_j,t)\rangle &= (\hat{\sigma}_j^{+}|\phi_0\rangle\langle\phi_0|\hat{\sigma}_j)|\Psi(t)\rangle \\ &= \textstyle\sum_m^{N+n_{\text{mode}}} c_m(t)\beta_j^m\hat{\sigma}_j^{+}|\phi_0\rangle \end{aligned} \quad (21)$$

with β_j^m the expansion coefficient of the excitonic basis state $\sigma_j^+|\phi_0\rangle$ in polaritonic state $|\psi^m\rangle$ (Equation 14), $c_m(t)$ the time-dependent expansion coefficients of the total wavefunction $|\Psi(t)\rangle$ (Equation 15), and $|\phi_0\rangle$ the ground state of the molecule-cavity system with no excitations of neither the molecules nor cavity modes (Equation 7).

The cavity mode excitations are described as plane waves that are delocalized in real space. We therefore obtained the amplitude of the cavity mode excitations in polaritonic eigenstate $|\psi^m\rangle$ at position z_j by Fourier transforming the projection of the cavity mode Fock states, in which cavity mode p is excited, onto $|\psi^m\rangle$:

$$\begin{aligned} |\psi^m_{\text{pho}}(z_j)\rangle &= \mathcal{FT}^{-1}\left[\sum_p^{n_{\text{mode}}}(\hat{a}_p^\dagger|\phi_0\rangle\langle\phi_0|\hat{a}_p)|\psi^m\rangle\right] \\ &= \frac{1}{\sqrt{N}}\sum_p^{n_{\text{mode}}}\alpha_p^m e^{i2\pi z_j p}\hat{a}_p^\dagger|\phi_0\rangle \end{aligned} \tag{22}$$

where α_p^m is the expansion coefficient of the cavity mode excitation $\alpha_p^\dagger|\phi_0\rangle$ in polaritonic state $|\psi^m\rangle$ (Equation 14) and we normalized by $1/\sqrt{N}$ rather than $1/\sqrt{L_z}$, as we represent the density on the grid of molecular positions. The total contribution of the cavity mode excitations to the wavepacket at position z_j at time t was then obtained as the weighted sum over the Fourier transforms:

$$\begin{aligned} |\Psi^{\text{pho}}(z_j,t)\rangle &= \sum_m^{N+n_{\text{mode}}} c_m(t)\times\mathcal{FT}^{-1}\left[\sum_p^{n_{\text{mode}}}(\hat{a}_p^\dagger|\phi_0\rangle\langle\phi_0|\hat{a}_p)|\psi^m\rangle\right] \\ &= \sum_m^{N+n_{\text{mode}}} c_m(t)\frac{1}{\sqrt{N}}\sum_p^{n_{\text{mode}}}\alpha_p^m e^{i2\pi z_j p}\hat{a}_p^\dagger|\phi_0\rangle \end{aligned} \tag{23}$$

with $c_m(t)$ the time-dependent expansion coefficient of the adiabatic polaritonic state $|\psi^m\rangle$ in the total wave function $|\Psi(t)\rangle$ (Equation 15).

3.3 Transient transmission

Under the assumption that we can neglect reflection, the transmission of light through the system, T, is related to absorbance, A, via the Lambert-Beer law:

$$-\ln(T) = A = \varepsilon_{\mathrm{a}} C d \tag{24}$$

with ε_{a} the absorption coefficient, C the concentration of absorbers (in our case the Rhodamines), and d the length of the path through which the light passes. Based on the article of Pandya and co-workers,[21] we assume that they probed how the transmission of photons with an energy equal to the excitation from the total ground state $|\phi_0\rangle$ (Equation 7, *i.e.*, no excitation in neither the molecules, nor cavity) into the LP branch at 640 nm for their BODIPY-R cavity systems, changes as a function of time (t) and position (here z) after the interaction of the molecule-cavity system with the pump pulse. Assuming furthermore that excitation from the LP or UP into the two-photon manifold is negligible due to absence of resonant transitions at that wavelength, the absorbance of the sample at position z and time t is proportional to the concentration of unexcited molecules:

$$C(z,t) = C_0 - |\Psi(z,t)|^2 \tag{25}$$

with C_0 the concentration of the Rhodamine molecules in the cavity *before* interaction with the pump-pulse. We assume that C_0 is uniform and homogeneous. With these approximations, the

transient normalized differential transmission in our simulations was calculated as:

$$\begin{aligned}\frac{\Delta T(z,t)}{T_0} &= \frac{T(z,t)-T_0}{T_0} \\ &= \frac{e^{-\varepsilon_{\rm a}dC(z,t)}-e^{-\varepsilon_{\rm a}dC_0}}{e^{-\varepsilon_{\rm a}dC_0}} \\ &= \frac{e^{-\varepsilon_{\rm a}d(C_0-|\Psi(z,t)|^2)}-e^{-\varepsilon_{\rm a}dC_0}}{e^{-\varepsilon_{\rm a}dC_0}} \\ &= e^{\varepsilon_{\rm a}d|\Psi(z,t)|^2}-1\end{aligned} \tag{26}$$

In analogy to Equation S4 in the Supporting Information of the work by Balasubrahmanyam *et al.*[?], the mean squared displacement (MSD) of the transient transmisson signal $\Delta T/T_0$ was calculated as:

$$\mathrm{MSD}_T = \sum_i^N (z_i - z_0)^2 \frac{\Delta T(z_i,t)}{T_0} = \sum_i^N (z_i - z_0)^2 \left[e^{\varepsilon_{\rm a}d|\Psi(z_i,t)|^2}-1\right] \tag{27}$$

where the sum runs over the positions, z_i of all molecules, and z_0 is the average position of the wavepacket at the start of the simulation. We treated $\varepsilon_{\rm a}d$ as a single parameter between 0 and 1. In Figure S3, we plot the MSD_T of the transmitted signal for various values of $\varepsilon_{\rm a}d$. Based on the similarity of the plots, we conclude that the results of the analysis are not very sensitive to the choice of this parameter.

3.4 Estimation of the duration and propagation velocity of the ballistic phase

The propagation velocity, $\upsilon_{\rm bal}$, and duration, $\tau_{\rm bal}$, of the ballistic phase were obtained by fitting the model of Pandya *et al.* (Equation 5 in their paper[21]) without σ_0 (which is zero in our simulations) to the MSD_T of $\Delta T(z,t)/T_0$ from $t=0$ to $t=t_{\mathrm{MSD}_T^{\max}}$, when the $\mathrm{MSD_T}$ reaches its maximum:

$$\mathrm{MSD}_T(t) = \upsilon_{\rm bal}^2 t^2 \exp\left(-t/\tau_{\rm bal}\right) \tag{28}$$

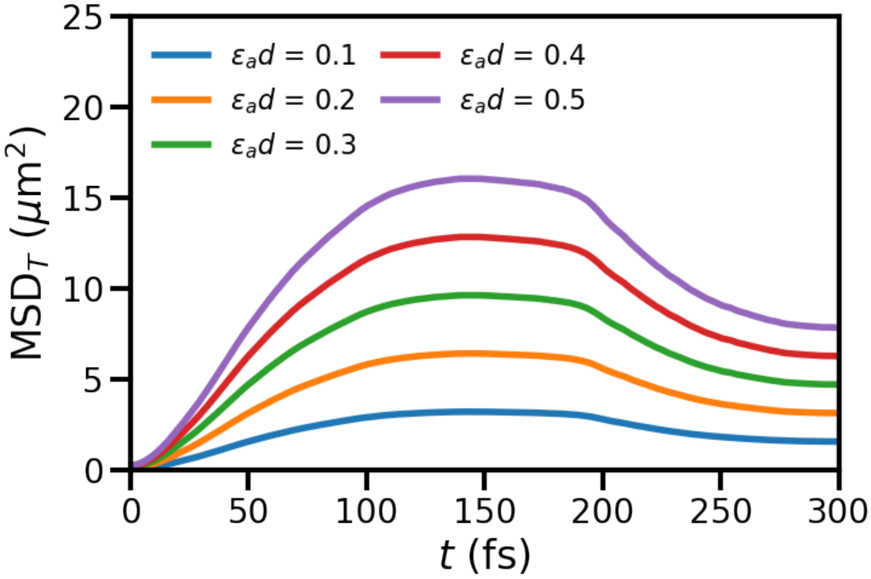

Figure S3: Mean squared displacement (MSD$_T$) of the $\Delta T(z,t)/T_0$ at time t after instantaneous excitation of a Gaussian wavepacket of UP states in a cavity with 512 molecules and a lifetime of $\tau_{\text{cav}} = 60$ fs, plotted for various values of $\epsilon_{\text{a}}d$.

In Figure S4**a** these fits are plotted as dashed lines. The good agreement between the fit and the MSD$_T$ suggests that this function captures the initial MSD$_T$ as a function of time for all cavities.

The propagation velocity, v_{bal}, only weakly depends on the cavity lifetime, τ_{cav} (Figure S4**b**), because transport in the ballistic regime is primarily governed by the group velocities of UP states. The duration of the ballistic regime, τ_{bal}, however, depends more strongly on the cavity lifetime (Figure 4**b** in the main text), suggesting that the increase of propagation distance with cavity lifetime is mainly due to a longer duration of the ballistic phase.

The error bars in the plots of v_{bal} (Figure S4**b**) and τ_{bal} (Figure 4**b** in the main text) correspond to the standard deviation σ_x of the $S = 5$ trajectories for each cavity mode lifetime:

$$\sigma_x = \sqrt{\frac{\sum_{i=1}^{S} (x_i - \overline{x})^2}{S - 1}} \tag{29}$$

where x_i and $\overline{x}$ are, respectively, the values of v_{gr} or τ_{bal}, and their averages, respectively.

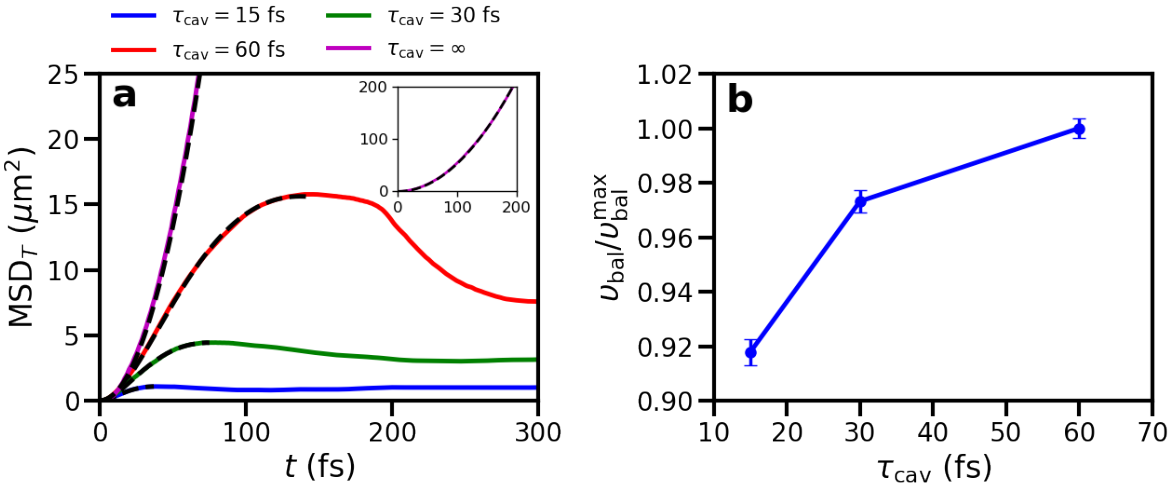

Figure S4: Panel **a**: MSD_T of the transient differential transmission after excitation in the perfect cavity (purple) and in cavities with the lifetimes τ_{cav} of 60 fs (red), 30 fs (green), and 15 fs (blue). The inset shows MSD_T of the perfect cavity during 200 fs of simulations. Panel **b**: Normalised average polariton group velocity υ_{gr} as a function of the cavity lifetime, extracted from a quadratic fit to MSD_Ts (Equation 28).

3.5 Estimation of the diffusion coefficient in the diffusive phase

As explained in the main text, we extracted the diffusion coefficient from the wavepacket MSD. We consider only the part of the wavepacket that is moving slower than the maximum group velocity of the LP (υ_{LP}^{max}, Figure S5) and performed a linear fit to the last 100 fs of the trajectories:

$$MSD_{diff}(t) = 2D \cdot t \tag{30}$$

These diffusion coefficients are plotted as a function of cavity lifetime in Figure 4**c** of the main text.

3.6 Influence of cavity decay rate on the Rabi splitting

A coupled harmonic oscillator model was employed to explore how the value of Rabi splitting changes with the cavity decay rate. The polaritonic dispersion was calculated by diagonalizing the Tavis-Cummings Hamiltonian (equation 4) with the decay rate $-\hbar\gamma_{cav}/2$ added to the energies of the cavity modes. The Rabi splitting was then obtained as a minimal energy difference between the

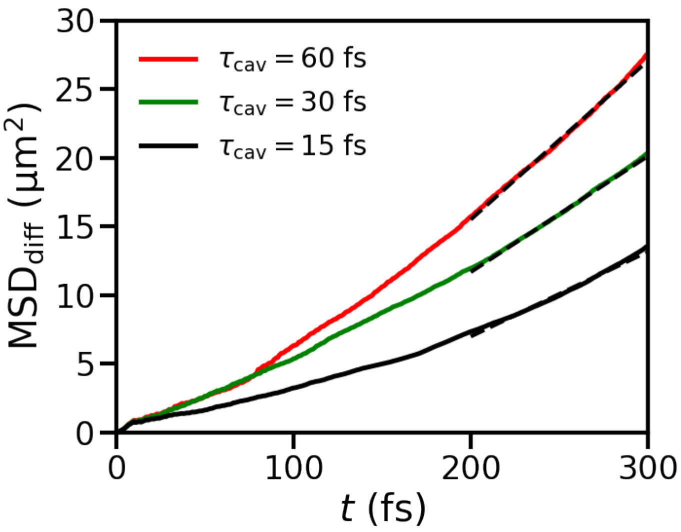

Figure S5: Mean squared displacement of the diffusive part of the total polariton wave function $|\Psi(t)|^2$ as a function of time in cavities with decay rates τ_{cav} of 60 fs (red), 30 fs (green), and 15 fs (black). The dashed lines are linear fits to the last 100 fs of the MSDs.

upper and lower polariton branches, which can also be analytically expressed as[22,23]

$$\Omega^{\text{Rabi}} = 2\sqrt{g_N^2 - \frac{\gamma_{\text{cav}}^2}{16}} \tag{31}$$

with $g_N = g\sqrt{N}$ the *collective* coupling strength when N molecules are coupled to the cavity mode.

Equation 31 suggests that the Rabi splitting turns to zero when $\gamma_{\text{cav}} = 4g_N$. Indeed, as Figure S6**a** shows, the Rabi splitting vanishes at $\gamma_{\text{cav}} = 650$ meV in the system with $N = 256$ molecules and $g = 10.15625$ meV, which gives $g_N = 162.5$ meV. Despite the influence of the cavity Q-factor on the Rabi splitting and, hence, the group velocity of polaritons (Figure S6**b**), this effect is very modest, as shown in the inset of Figure S6. Therefore, for the cavity lifetimes considered in our simulations, the variation of the Rabi splitting and polariton group velocities is negligible.

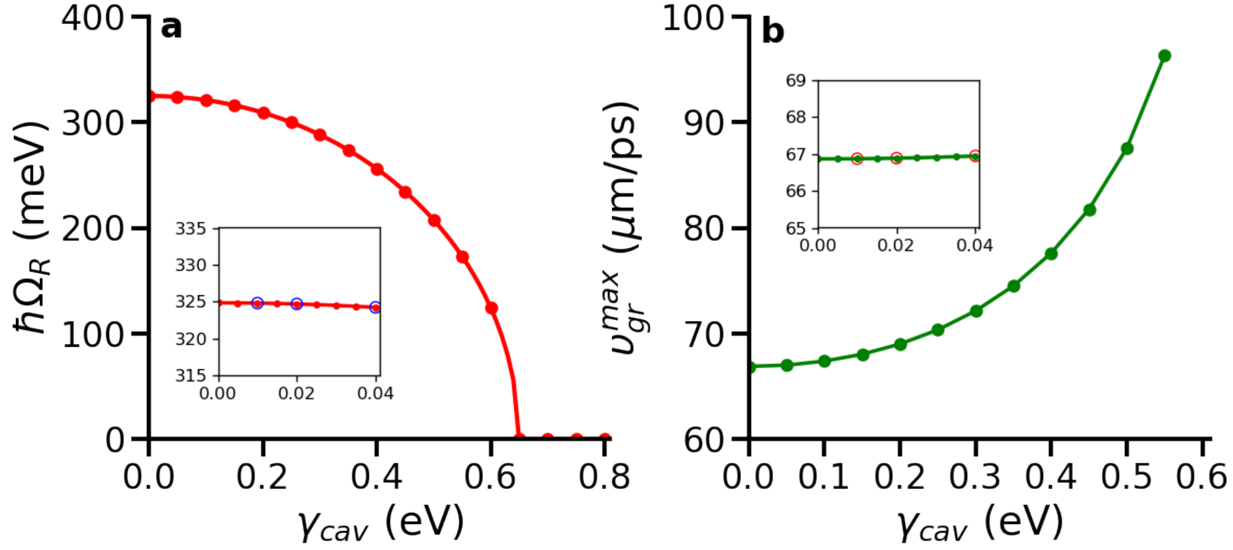

Figure S6: Rabi splitting (panel **a**) and the maximum group velocity of the LP (panel **b**) as a function of the cavity decay rate, $\gamma_{\rm cav}$. The insets show the initial trend of the curve with circles indicating the values corresponding to the decay rates considered in the current study.

4 Additional simulations

4.1 Simulations with different cavity detunings

To explore the effect of the cavity detuning, *i.e.*, the energy difference between the fundamental cavity mode at $k_z = 0$ and the molecular absorption maximum, on the transport of polaritons in our model, we performed additional simulations with exciton-photon detunings of 270 meV and 470 meV. All other parameters, including the cavity decay rates ($\gamma_{\rm cav} = 33.7$ ps^{-1}) and the energy bandwidth of the excitation, were kept the same. The results of these simulations, in particular the mean squared displacement (Figure S7), suggest that changing the cavity mode detuning by ~ 100meVs has very little impact on the wavepacket propagation under the resonant broad-band excitation conditions employed in this work. Indeed, for all detunings, we observe a transition from ballistic propagation into diffusion as well as a contraction of the wavepacket around 75 fs.

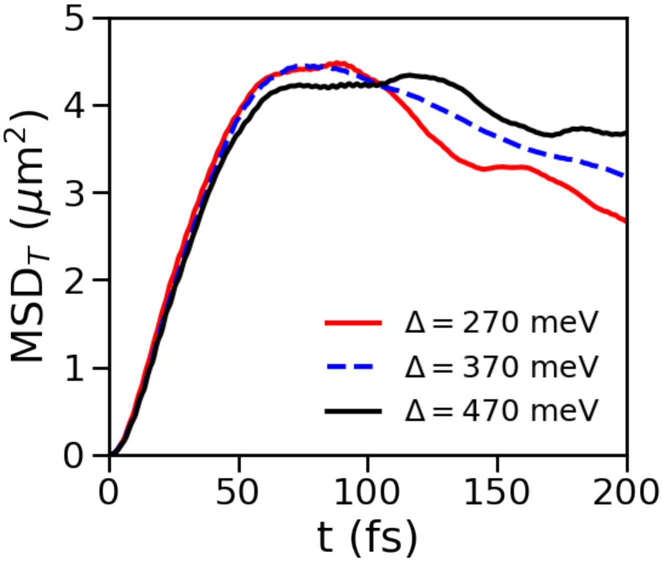

Figure S7: MSD$_T$ of the wavepacket for three detunings Δ of the fundamental cavity mode at $k_z = 0$: $\Delta = 270$ meV (solid red line), 370 meV (dashed blue line), and 470 meV (solid black line). The plots were obtained for the average data of five simulations with $\Delta = 370$ meV and for a single run with $\Delta = 270$ and 470 meV.

4.2 Propagation of narrow wavepackets

To investigate the effect of both reducing the bandwidth of the excitation pulse to a smaller, but still finite value, and shifting the central energy of the wave packet, we performed three additional simulations initially exciting narrow Gaussian wavepackets ($\sigma = 0.4$ μm^{-1}), centred at $k_z = 5.03$ μm^{-1}, $k_z = 10.56$ μm^{-1}, and $k_z = 17.22$ μm^{-1} (Figure S8**a**). Varying the center of the initial wavepacket excitation as such corresponds to setting the energy gap ΔE_k between the center of this wavepacket and the center of the dark states manifold (*i.e.*, the absorption maximum of Rhodamine at 4.18 eV) equal to 78 meV, 256 meV, and 959 meV, respectively.

Figure S8**d**-**f**, displays the propagation of these three wavepackets as a function of both cavity length and simulation time. Because the rate of population transfer from UP states into the dark states manifold is inversely proportional to the energy gap,[4] a narrow wavepacket centered at high k_z, and hence with a large energy gap to the dark state manifold, propagates purely ballistically while decaying irreversibly due to its highly photonic character (Figure S8**b**,**c**). The same wavepacket centered at lower k_z, hence with a smaller energy gap, has its ballistic phase reduced but survives

longer not only because the irreversible loss rate is smaller due to its reduced photonic character but also because this population can relax more efficiently into dark states, where it is protected from radiative losses.[16]

Furthermore, because of the highly lossy cavities employed for these additional simulations ($\gamma_{\text{cav}} = 66.7\text{ps}^{-1}$), the radiative decay rate in combination with the rate of population transfers from the bright UP states into the dark states, causes a fast depletion of the initially populated UP states such that wavepacket contraction occurs earlier as the photonic character and the energy gap to the dark state manifold increases (Figure S8**c**).

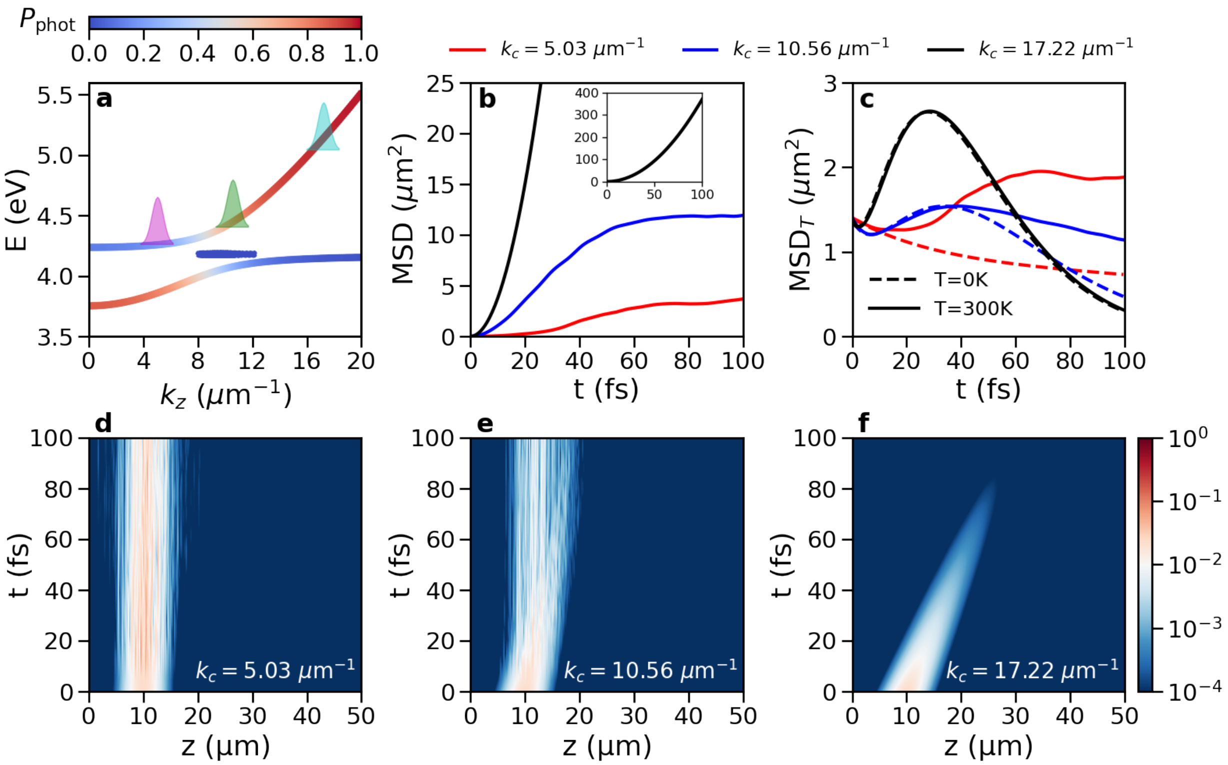

Figure S8: Propagation of narrow polariton wave packets centred at $k_z = 5.03$, 10.56 and 17.22 μm^{-1}. The bandwidth of all packets was set to 0.42 μm^{-1}. Panel **a**: Dispersion of polaritons. The total contribution of all cavity modes to each polaritonic state (P_{phot}) is indicated by colors. The pink, green and cyan bells schematically illustrate the initial location of the wave packets. Panels **b** and **c**: MSD and MSD_T of the transient differential transmission of the three wave packets. As a reference, we also plot the MSD_T of the transmission at T=0 K in panel **c** (dashed lines). Panels **d**-**f**: Probability density of the total wave function, $|\Psi(t)|^2$, as a function of distance (horizontal axis) and time (vertical axis) after resonantly exciting a narrow wavepacket of states in the UP branch centered at $k_z = 5.03$ (**d**), 10.56 (**e**) and 17.22 μm^{-1}(**f**).

References

(1) Jaynes, E. T.; Cummings, F. W. Comparison of quantum and semiclassical radiation theories with to the beam maser. *Proc. IEEE* **1963**, *51*, 89–109.

(2) Tavis, M.; Cummings, F. W. Approximate solutions for an N-molecule radiation-field Hamiltonian. *Phys. Rev.* **1969**, *188*, 692–695.

(3) Luk, H.-L.; Feist, J.; Toppari, J. J.; Groenhof, G. Multiscale Molecular Dynamics Simulations of Polaritonic Chemistry. *J. Chem. Theory Comput.* **2017**, *13*, 4324–4335.

(4) Tichauer, R.; Feist, J.; Groenhof, G. Multi-scale Dynamics Simulations of Molecular Polaritons: the Effect of Multiple Cavity Modes on Polariton Relaxation. *J. Chem. Phys* **2021**, *154*, 104112.

(5) Warshel, A.; Levitt, M. Theoretical studies of enzymatic reactions: Dielectric, electrostatic and steric stabilization of carbonium ion in the reaction of lysozyme. *J. Mol. Biol.* **1976**, *103*, 227–249.

(6) Boggio-Pasqua, M.; Burmeister, C. F.; Robb, M. A.; Groenhof, G. Photochemical reactions in biological systems: probing the effect of the environment by means of hybrid quantum chemistry/molecular mechanics simulations. *Phys. Chem. Chem. Phys.* **2012**, *14*, 7912–7928.

(7) Michetti, P.; Rocca, G. C. L. Polariton states in disordered organic microcavities. *Phys. Rev. B.* **2005**, *71*, 115320.

(8) Agranovich, V.; Gartstein, Y. Nature and Dynamics of Low-Energy Exciton Polaritons in Semiconductor Microcavities. *Phys. Rev. B* **2007**, *75*, 075302.

(9) Granucci, G.; Persico, M.; Toniolo, A. Direct semiclassical simulation of photochemical processes with semiempirical wave functions. *J. Chem. Phys.* **2001**, *114*, 10608–10615.

(10) Duan, Y.; Wu, C.; Chowdhury, S.; Lee, M. C.; Xiong, G. M.; Zhang, W.; Yang, R.; Cieplak, P.; Luo, R.; Lee, T.; Caldwell, J.; Wang, J. M.; Kollman, P. A point-charge force field for molecular mechanics simulations of proteins based on condensed-phase quantum mechanical calculations. *J. Comput. Chem.* **2003**, *24*, 1999–2012.

(11) Jorgensen, W. L.; Chandrasekhar, J.; Madura, J. D.; Impey, R. W.; Klein, M. L. Comparison of simple potential functions for simulatin liquid water. *J. Chem. Phys.* **1983**, *79*, 926–935.

(12) Berendsen, H.; Postma, J.; van Gunsteren, W.; la, A. D.; Haak, J. Molecular dynamics with coupling to an external bath. *J. Chem. Phys.* **1984**, *81*, 3684–3690.

(13) Hess, B.; Bekker, H.; Berendsen, H. J. C.; Fraaije, J. G. E. M. LINCS: A linear constraint solver for molecular simulations. *J. Comput. Chem.* **1997**, *18*, 1463–1472.

(14) Miyamoto, S.; Kollman, P. A. SETTLE: An analytical version of the SHAKE and RATTLE algorithms for rigid water molecules. *J. Comp. Chem.* **1992**, *18*, 1463–1472.

(15) Essmann, U.; Perera, L.; Berkowitz, M. L.; Darden, T.; Lee, H.; Pedersen, L. G. A smooth particle mesh Ewald potential. *J. Chem. Phys* **1995**, *103*, 8577–8592.

(16) Groenhof, G.; Climent, C.; Feist, J.; Morozov, D.; Toppari, J. J. Tracking Polariton Relaxation with Multiscale Molecular Dynamics Simulations. *J. Chem. Phys. Lett.* **2019**, *10*, 5476–5483.

(17) Groenhof, G.; Bouxin-Cademartory, M.; Hess, B.; de Visser, S. P.; Berendsen, H. J. C.; Olivucci, M.; A. E. Mark, M. A. R. Photoactivation of the photoactive yellow protein: Why photon absorption triggers a trans-to-cis isomerization of the chromophore in the protein. *J. Am. Chem. Soc* **2004**, *124*, 4228–4232.

(18) Hess, B.; Kutzner, C.; van der Spoel, D.; Lindahl, E. GROMACS 4: Algorithms for Highly Efficient, Load-Balanced, and Scalable Molecular Simulation. *J. Chem. Theory Comput.* **2008**, *4*, 435–447.

(19) Ufimtsev, I.; Martínez, T. J. Quantum Chemistry on Graphical Processing Units. 3. Analytical Energy Gradients and First Principles Molecular Dynamics. *J. Chem. Theory Comput.* **2009**, *5*, 2619–2628.

(20) Titov, A.; Ufimtsev, I.; Luehr, N.; Martínez, T. J. Generating Efficient Quantum Chemistry Codes for Novel Architectures. *J. Chem. Theory Comput.* **2013**, *9*, 213–221.

(21) Pandya, R.; Ashoka, A.; Georgiou, K.; Sung, J.; Jayaprakash, R.; Renken, S.; Gai, L.; Shen, Z.; Rao, A.; Musser, A. J. Tuning the Coherent Propagation of Organic Exciton-Polaritons through Dark State Delocalization. *Adv. Sci.* **2022**, 2105569.

(22) Antoniou, P.; Suchanek, F.; Varner, J. F.; Foley IV, J. J. Role of Cavity Losses on Nonadiabatic Couplings and Dynamics in Polaritonic Chemistry. *J. Phys. Chem. Lett.* **2020**, *11*, 9063–9069.

(23) Tropf, L.; Dietrich, C. P.; Herbst, S.; Kanibolotsky, A. L.; Skabara, P. J.; Würthner, F.; Samuel, I. D. W.; Gather, M. C.; Hoefling, S. Influence of optical material properties on strong coupling in organic semiconductor based microcavities. *Appl. Phys. Lett.* **2017**, *110*, 153302.
